# Supplementary material for: 2,5-Bis(2,2,2-trifluoroethoxy)phenyl-tethered 1,3,4-Oxadiazoles Derivatives: Synthesis, In Silico Studies, and Biological Assessment as Potential Candidates for Anti-Cancer and Anti-Diabetic Agent
Source: Molecules. 2022 Dec 8;27(24):8694. doi: 10.3390/molecules27248694 (PMC9781914; doi:10.3390/molecules27248694)
Supplement: Supplementary file 1 [file molecules-27-08694-s001.zip › molecules-2029487-supplementary.pdf]

# Supplementary file

## 2,5-Bis(2,2,2-trifluoroethoxy)phenyl-tethered 1,3,4-Oxadiazoles Derivatives: Synthesis, In Silico Studies, and Biological Assessment as Potential Candidates for Anti-Cancer and Anti-Diabetic Agent

**Sathyanarayana D. Shankara [1,2], Arun M. Isloor [1,*], Avinash K. Kudva [3], Shamprasad Varija Raghu [4], Pavan K. Jayaswamy [5], Pushyaraga P. Venugopal [6], Praveenkumar Shetty [5,7] and Debashree Chakraborty [6]**

[1] Membrane and Separation Technology Laboratory, Department of Chemistry, National Institute of Technology Karnataka, Surathkal, Mangalore 575025, India

[2] Solara Active Pharma Sciences, No:120 A&B, Industrial Area, Baikampady, New Mangalore, Mangalore 575011, India

[3] Department of Biochemistry, Mangalore University, Mangalagangothri, Mangalore 574199, India

[4] Neurogenetics Lab, Department of Applied Zoology, Mangalore University, Mangalagangothri, Mangalore 574199, India

[5] Central Research Laboratory, KS. Hegde Medical Academy, Nitte (Deemed to be University), Deralakatte, Mangalore 575018, India

[6] Biophysical and Computational Chemistry Laboratory, Department of Chemistry, National Institute of Technology Karnataka, Surathkal, Mangalore 575025, India

[7] Department of Biochemistry, K.S. Hegde Medical Academy, Nitte (Deemed to be University), Deralakatte, Mangalore 575018, India

* Correspondence: isloor@yahoo.com; Fax: 91-824-2474033

**Supplementary file –** Spectral data (IR,$^{1}$H NMR,$^{13}$C NMR, MASS Spectrum) of synthesized compounds

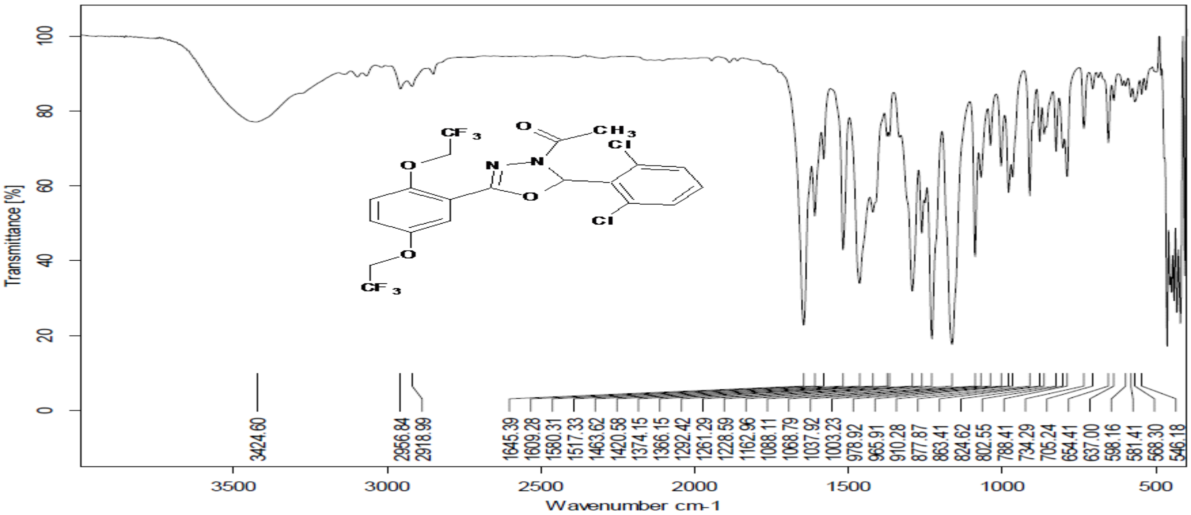


S.1: IR spectrum of 1-{5-[2,5-bis(2,2,2-trifluoroethoxy)phenyl]-2-(2,6-dichlorophenyl)-1,3,4-oxadiazol-3(2*H*)-yl}ethanone(5a)

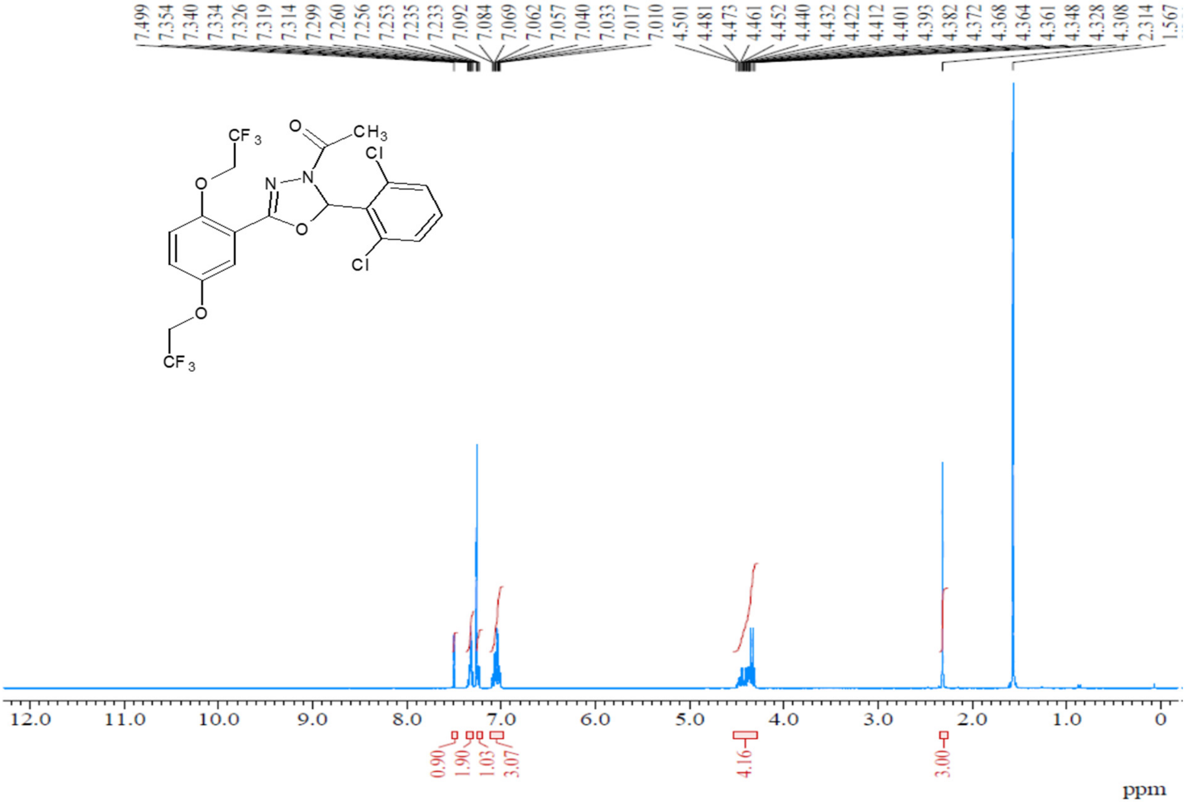


S.2: $^{1}$H NMR spectrum of 1-{5-[2,5-bis(2,2,2-trifluoroethoxy)phenyl]-2-(2,6-dichlorophenyl)-1,3,4-oxadiazol-3(2*H*)-yl}ethanone(5a)

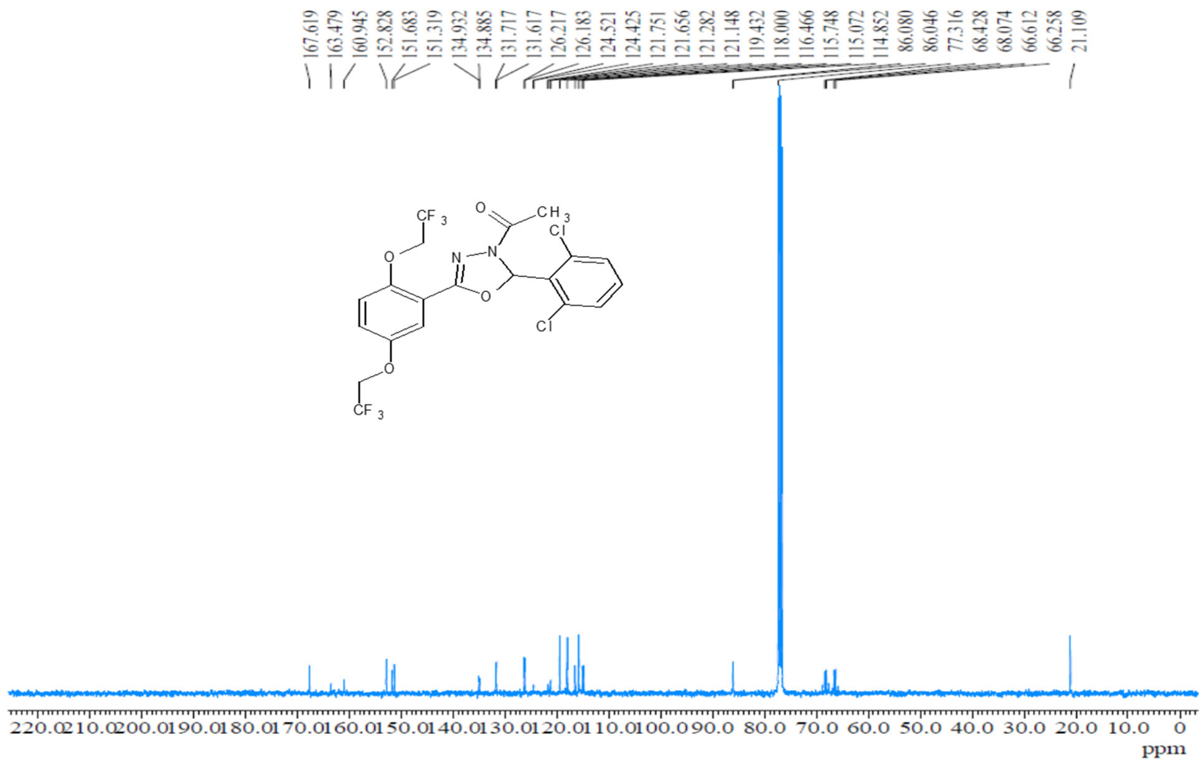

S.3: $^{13}$C NMR spectrum of 1-{5-[2,5-bis(2,2,2-trifluoroethoxy)phenyl]-2-(2,6-dichlorophenyl)-1,3,4-oxadiazol-3(2*H*)-yl}ethanone(5a)

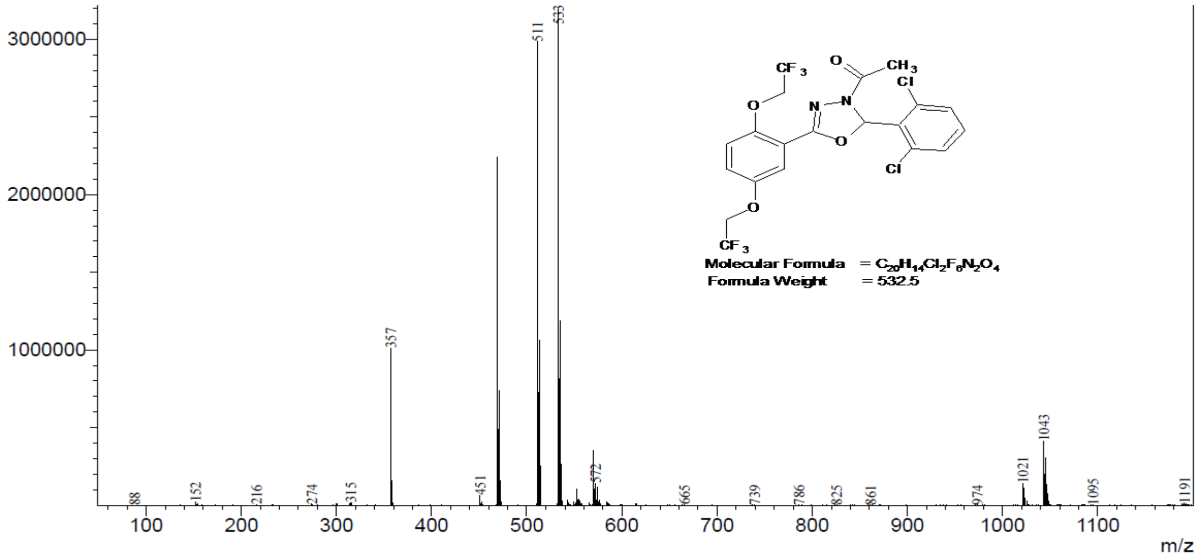


S.4: Mass spectrum of 1-{5-[2,5-bis(2,2,2-trifluoroethoxy)phenyl]-2-(2,6-dichlorophenyl)-1,3,4-oxadiazol-3(2*H*)-yl}ethanone(5a)

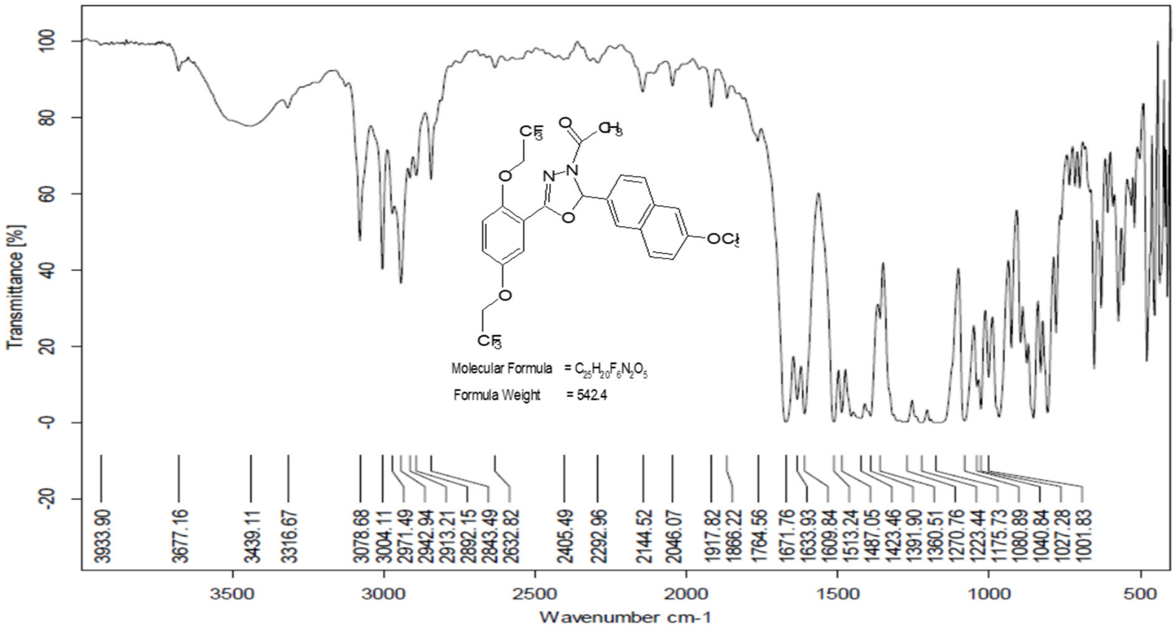


S.5: IR spectrum of 1-{5-[2,5-bis(2,2,2-trifluoroethoxy)phenyl]-2-(6-methoxy napthalyl)-1,3,4-oxadiazol-3(2*H*)-yl}ethanone(5b)

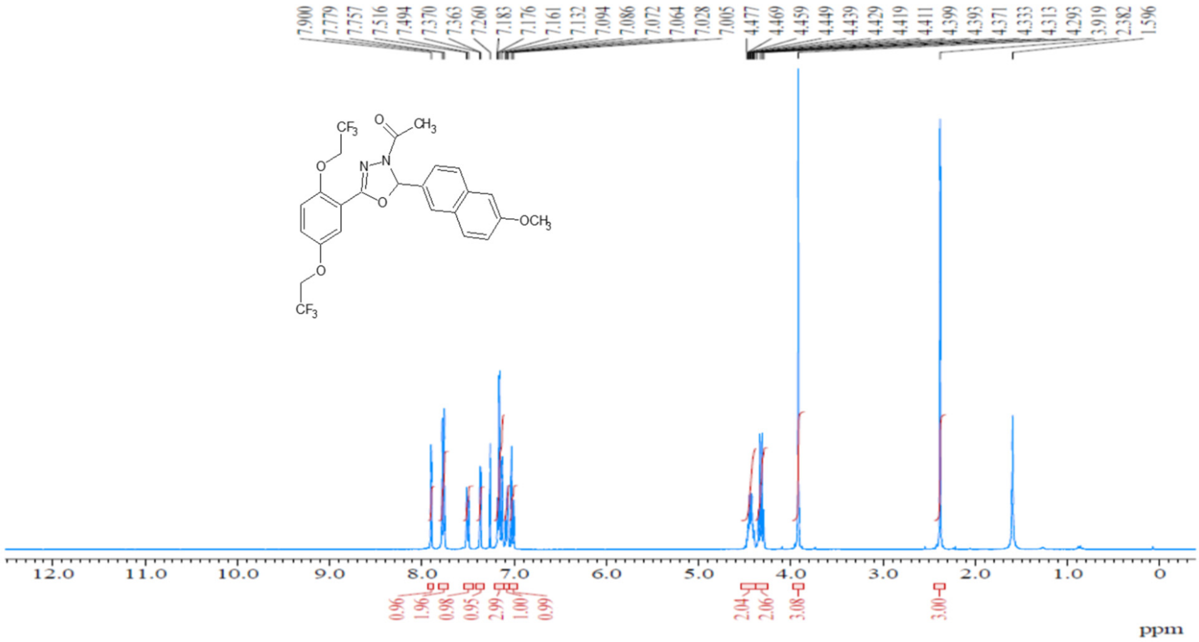


S.6: $^{1}$H NMR spectrum of 1-{5-[2,5-bis(2,2,2-trifluoroethoxy)phenyl]-2-(6-methoxy napthalyl)-1,3,4-oxadiazol-3(2*H*)-yl}ethanone(5b)

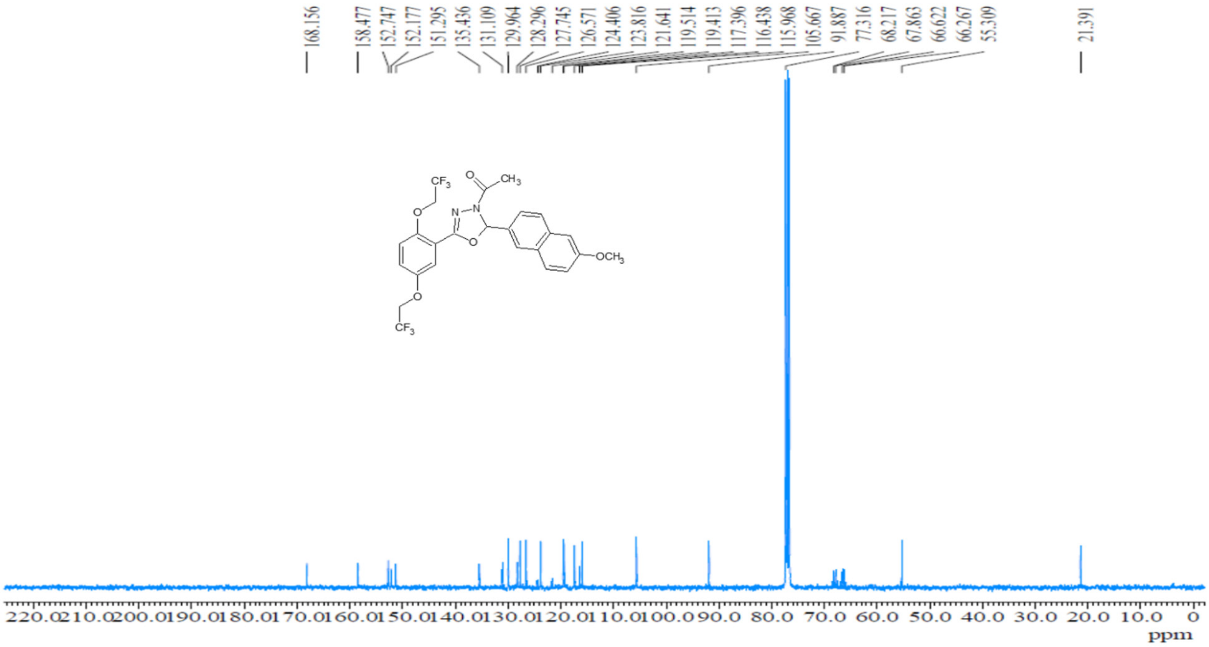


S.7: $^{13}$C NMR spectrum of 1-{5-[2,5-bis(2,2,2-trifluoroethoxy)phenyl]-2-(6-methoxy napthalyl)-1,3,4-oxadiazol-3(2*H*)-yl}ethanone(5b)

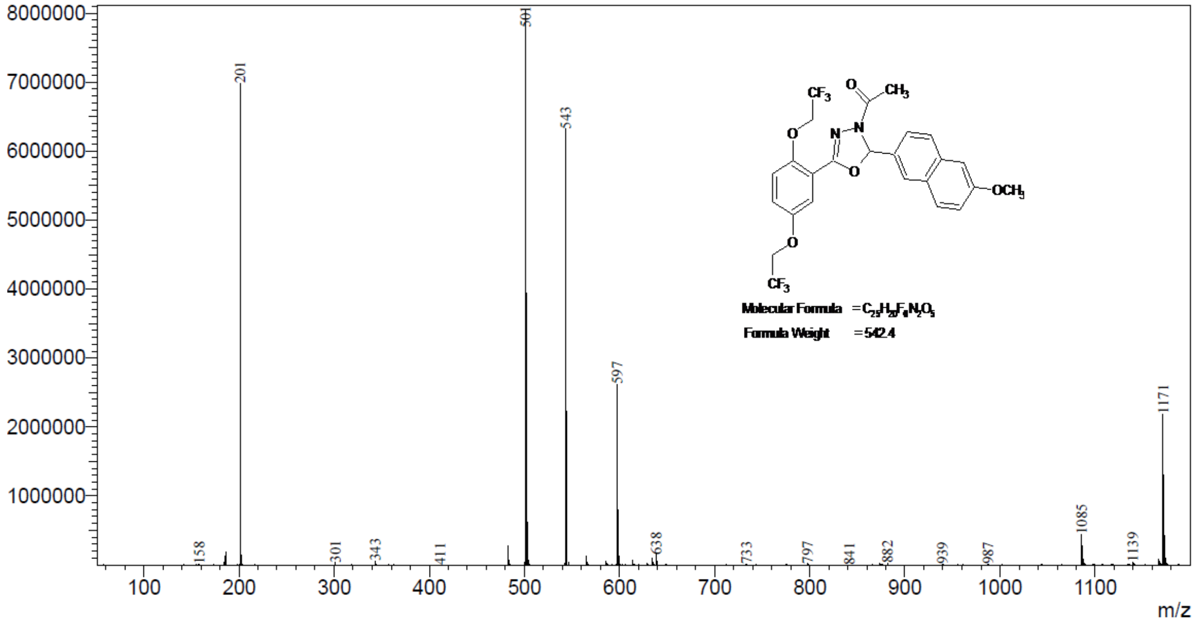


S.8: Mass spectrum of 1-{5-[2,5-bis(2,2,2-trifluoroethoxy)phenyl]-2-(6-methoxy napthalyl)-1,3,4-oxadiazol-3(2*H*)-yl}ethanone(5b)

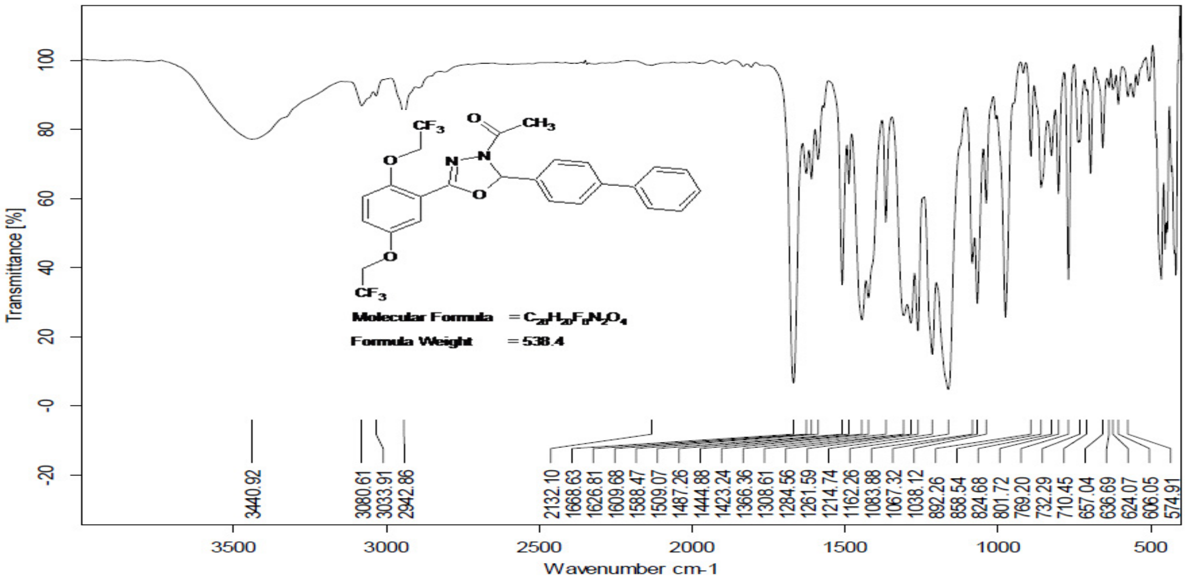


S.9: IR spectrum of 1-{5-[2,5-bis(2,2,2-trifluoroethoxy)phenyl]-2-(4-biphenyl)-1,3,4-oxadiazol-3(2*H*)-yl}ethanone(5c)

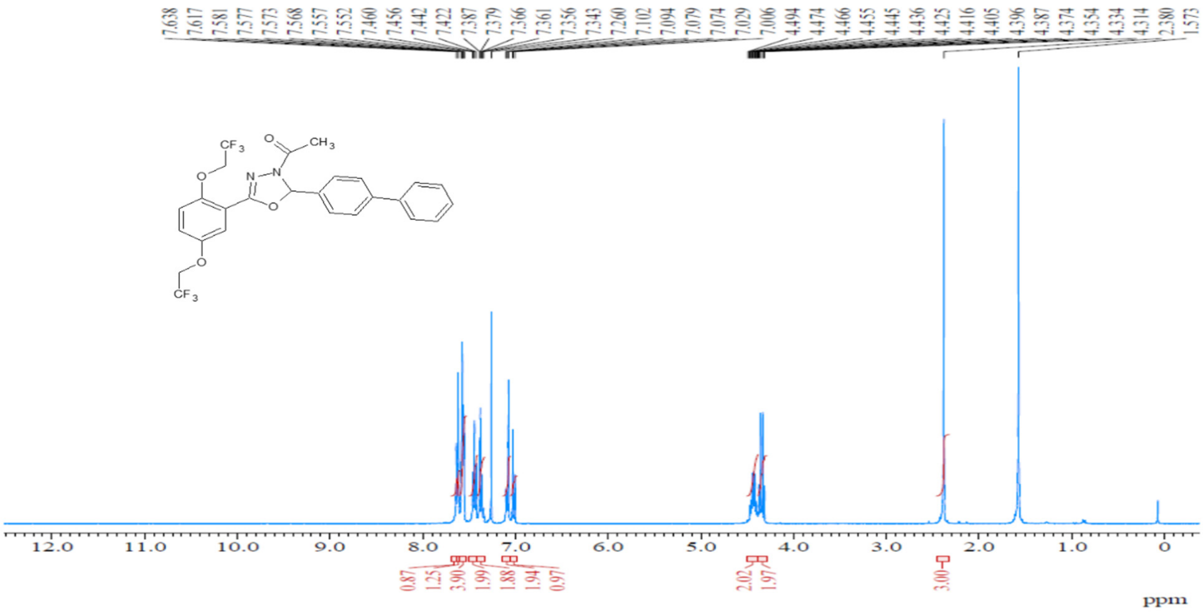


S.10: $^{1}$H NMR spectrum of 1-{5-[2,5-bis(2,2,2-trifluoroethoxy)phenyl]-2-(4-biphenyl)-1,3,4-oxadiazol-3(2*H*)-yl}ethanone(5c)

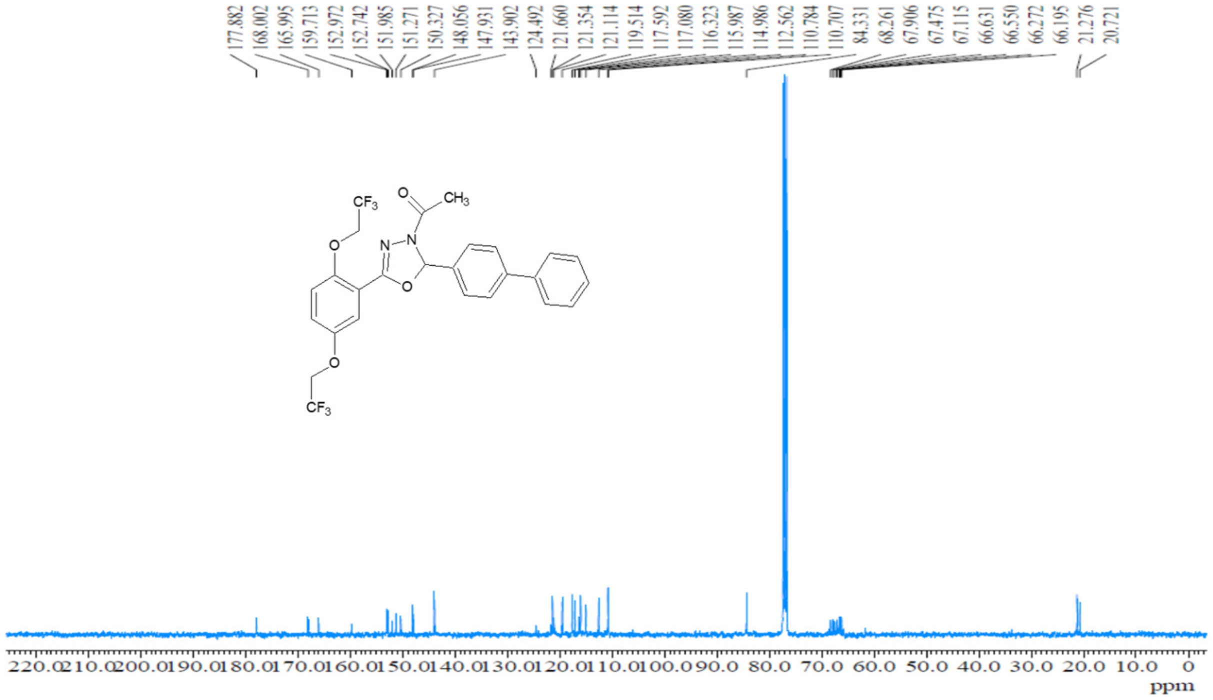


S.11: $^{13}$C NMR spectrum of 1-{5-[2,5-bis(2,2,2-trifluoroethoxy)phenyl]-2-(4-biphenyl)-1,3,4-oxadiazol-3(2*H*)-yl}ethanone(5c)

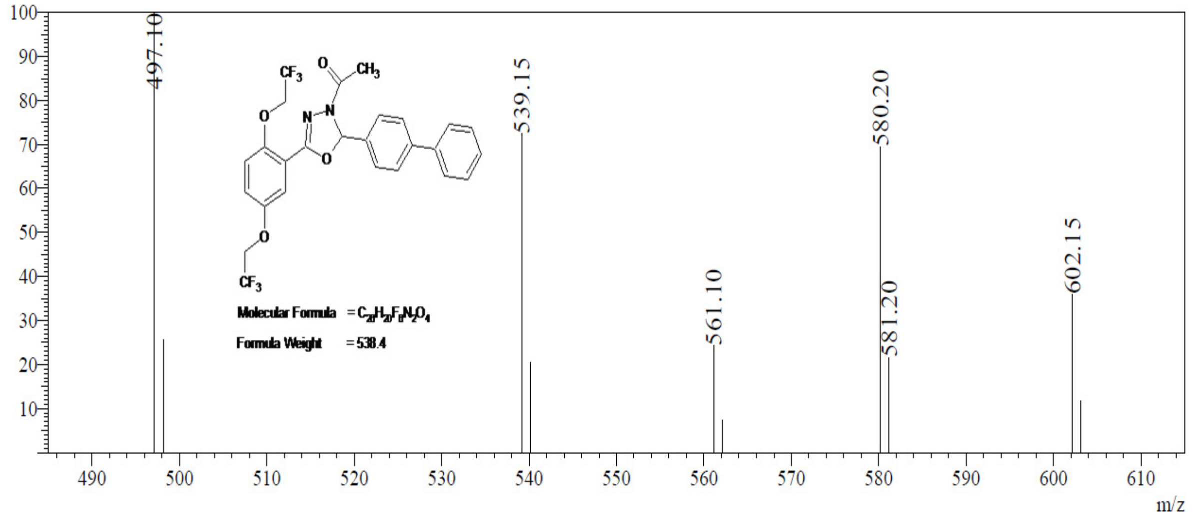


S.12: Mass spectrum of 1-{5-[2,5-bis(2,2,2-trifluoroethoxy)phenyl]-2-(4-biphenyl)-1,3,4-oxadiazol-3(2*H*)-yl}ethanone(5c)

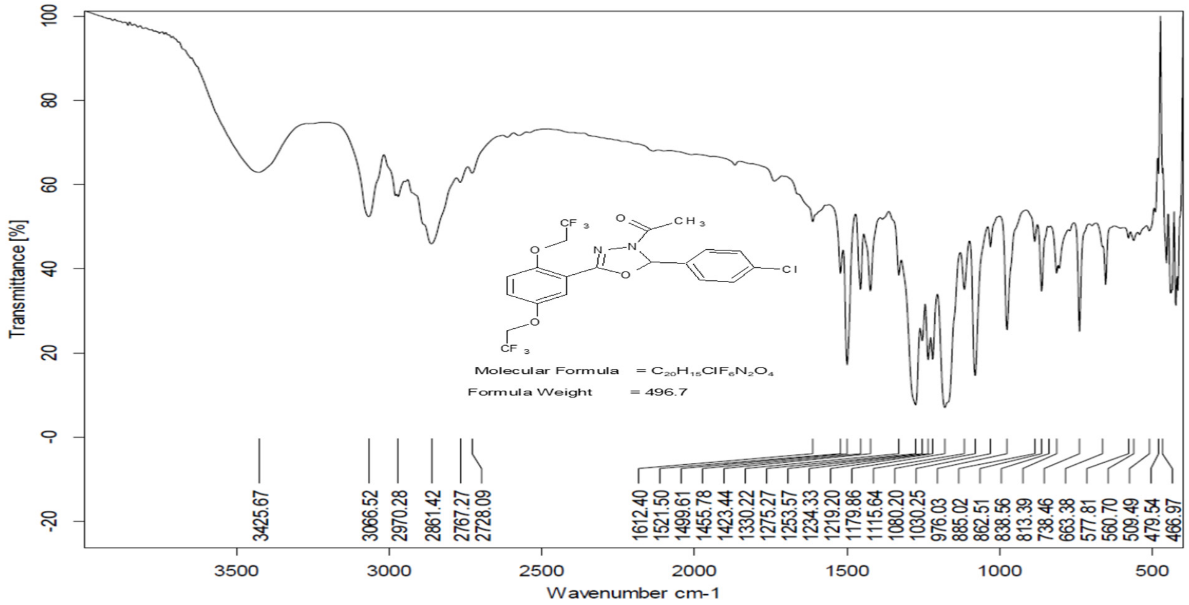


S.13: IR spectrum of 1-{5-[2,5-bis(2,2,2-trifluoroethoxy)phenyl]-2-(4-chloro phenyl)-1,3,4-oxadiazol-3(2*H*)-yl}ethanone(5d)

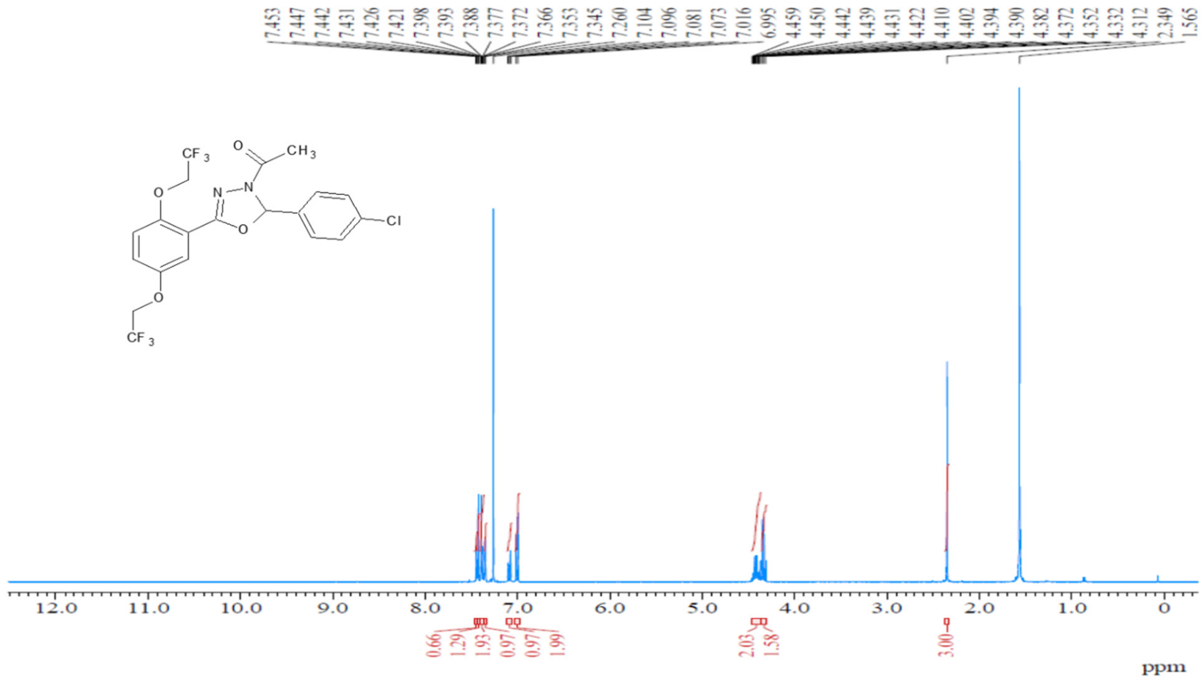


S.14: $^{1}$H NMR spectrum of 1-{5-[2,5-bis(2,2,2-trifluoroethoxy)phenyl]-2-(4-chloro phenyl)-1,3,4-oxadiazol-3(2*H*)-yl}ethanone(5d)

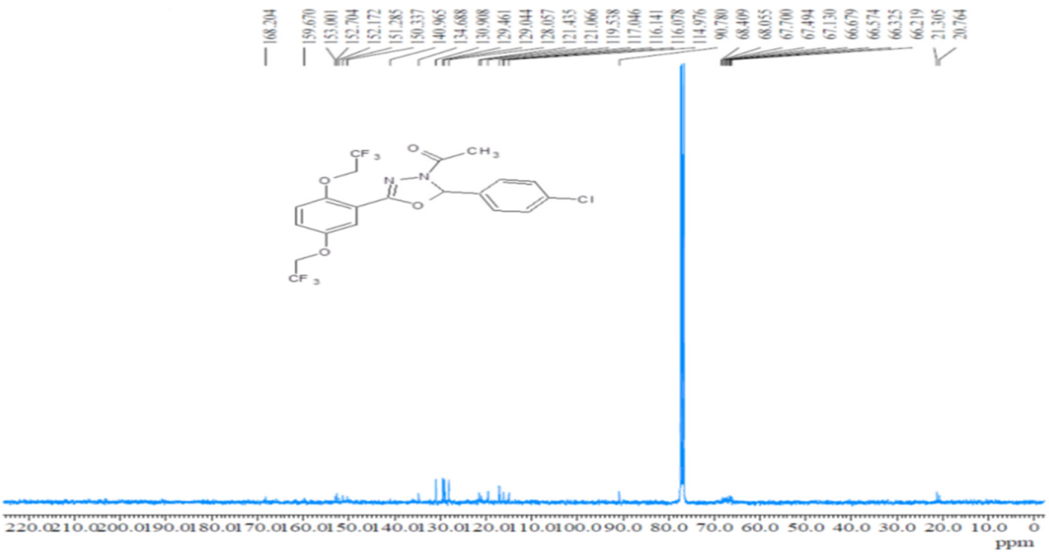


S.15: $^{13}$C NMR spectrum of 1-{5-[2,5-bis(2,2,2-trifluoroethoxy)phenyl]-2-(4-chloro phenyl)-1,3,4-oxadiazol-3(2*H*)-yl}ethanone(5d)

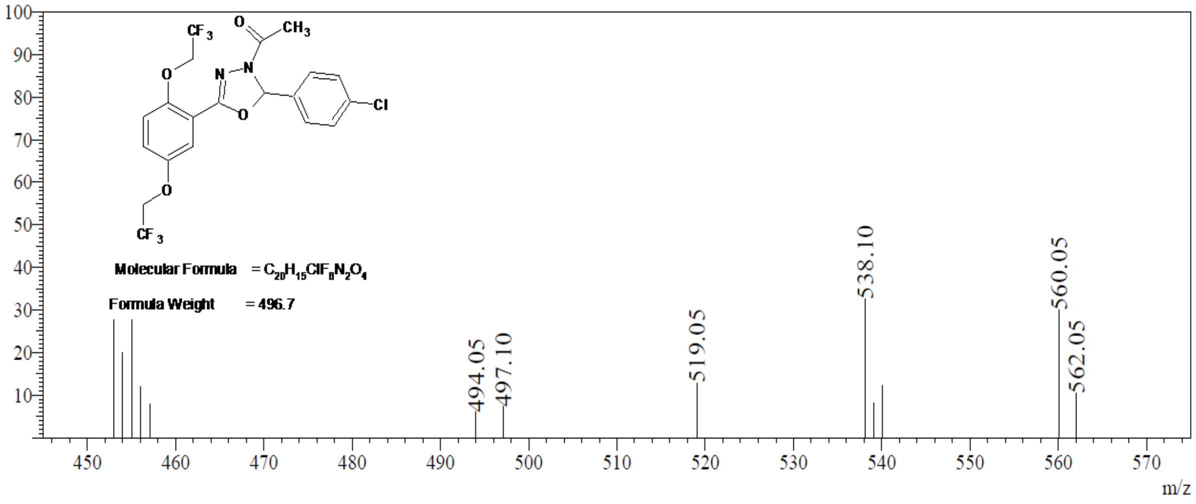


S.16: Mass spectrum of 1-{5-[2,5-bis(2,2,2-trifluoroethoxy)phenyl]-2-(4-chloro phenyl)-1,3,4-oxadiazol-3(2*H*)-yl}ethanone(5d)

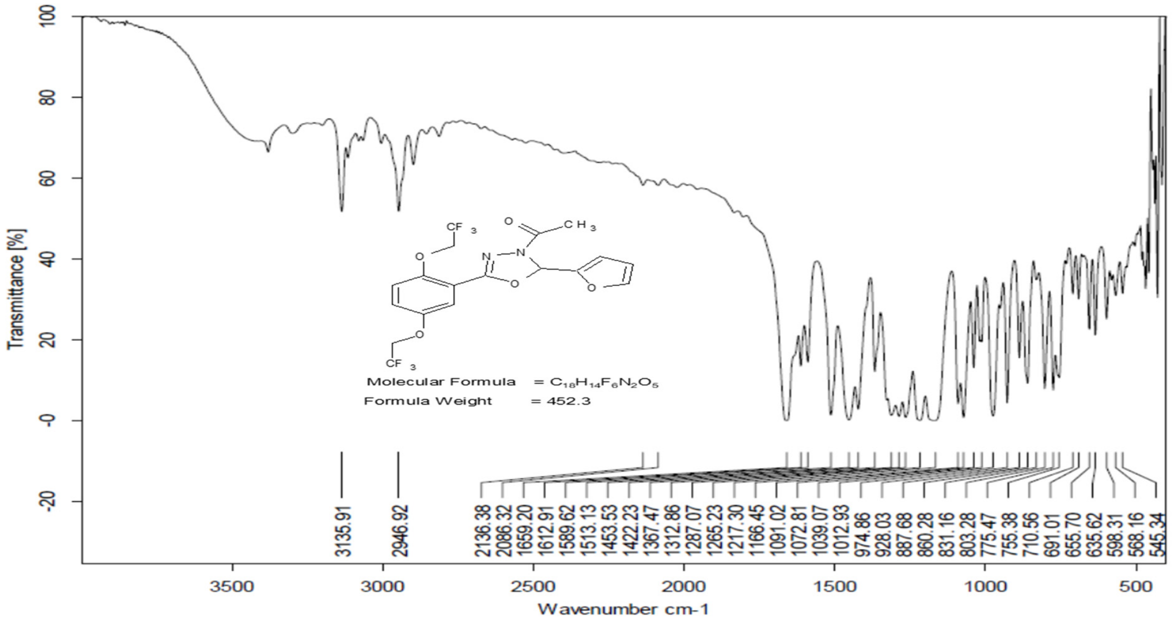


S.17: IR spectrum of of 1-{5-[2,5-bis(2,2,2-trifluoroethoxy)phenyl]-2-(furfuryl)-1,3,4-oxadiazol-3(2*H*)-yl}ethanone(5e)

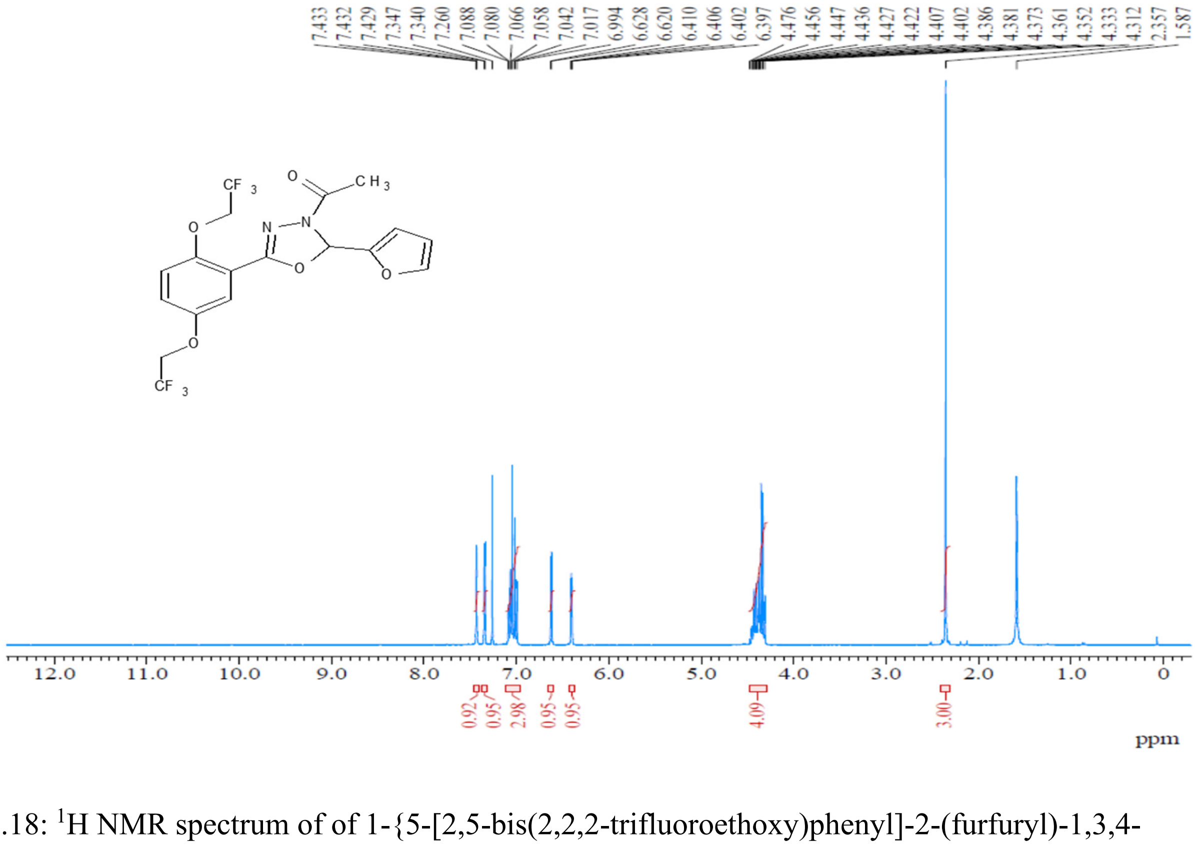


S.18: $^{1}$H NMR spectrum of of 1-{5-[2,5-bis(2,2,2-trifluoroethoxy)phenyl]-2-(furfuryl)-1,3,4-oxadiazol-3(2*H*)-yl}ethanone(5e)

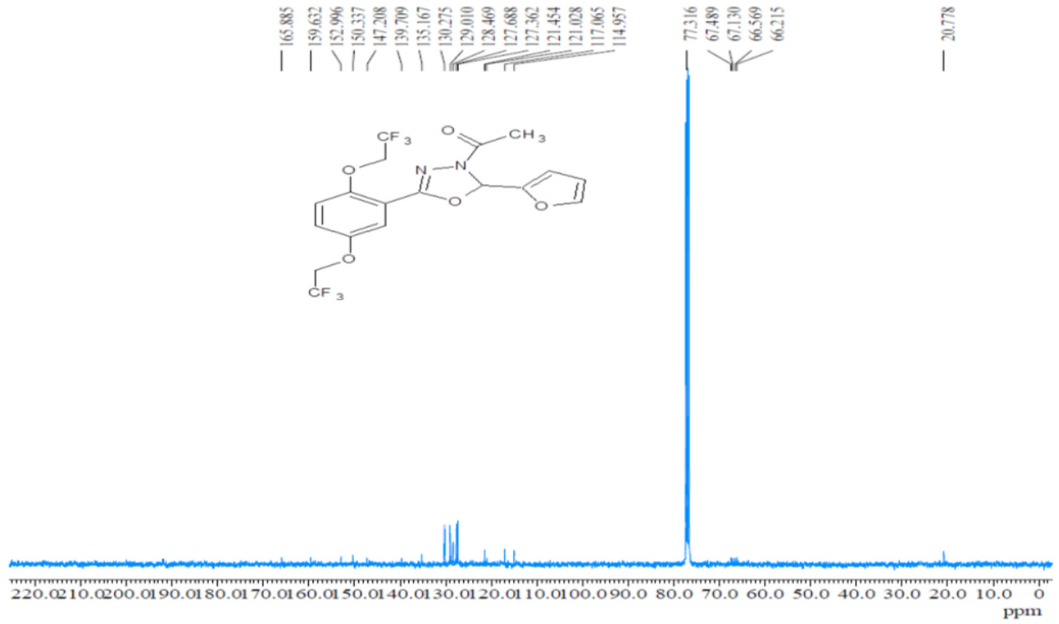


S.19: $^{13}$C NMR spectrum of of 1-{5-[2,5-bis(2,2,2-trifluoroethoxy)phenyl]-2-(furfuryl)-1,3,4-oxadiazol-3(2*H*)-yl}ethanone(5e)

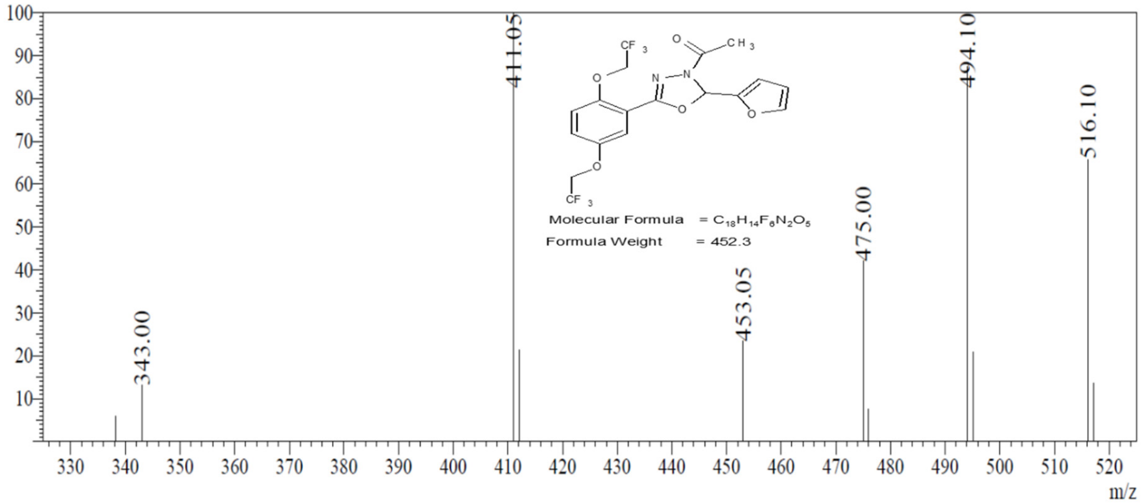


S.20: Mass spectrum of of 1-{5-[2,5-bis(2,2,2-trifluoroethoxy)phenyl]-2-(furfuryl)-1,3,4-oxadiazol-3(2*H*)-yl}ethanone(5e)

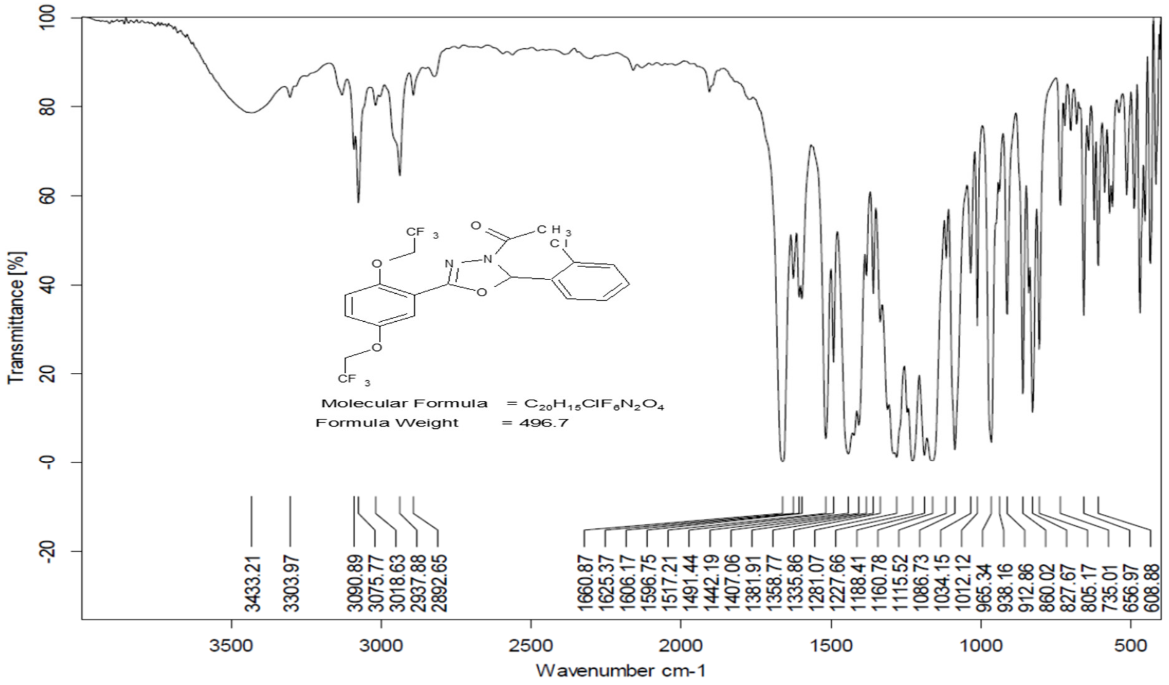


S.21: IR spectrum of of 1-{5-[2,5-bis(2,2,2-trifluoroethoxy)phenyl]-2-(2-chloro phenyl)-1,3,4-oxadiazol-3(2*H*)-yl}ethanone(5f)

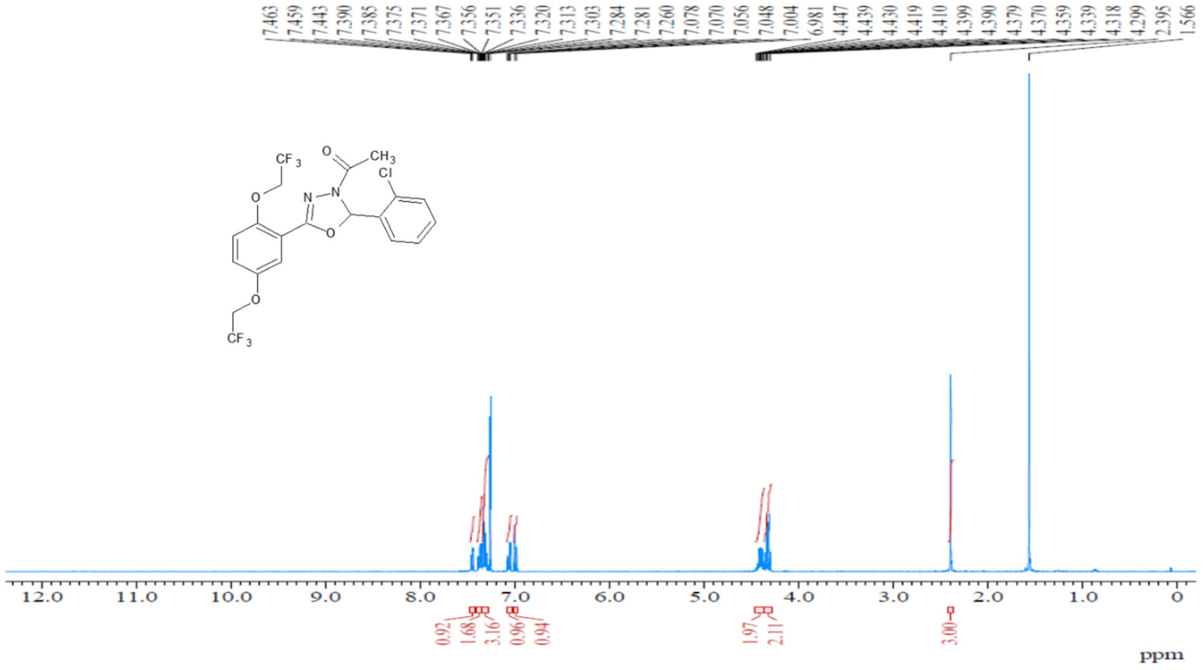


S.22: $^{1}$H NMR spectrum of of 1-{5-[2,5-bis(2,2,2-trifluoroethoxy)phenyl]-2-(2-chloro phenyl)-1,3,4-oxadiazol-3(2*H*)-yl}ethanone(5f)

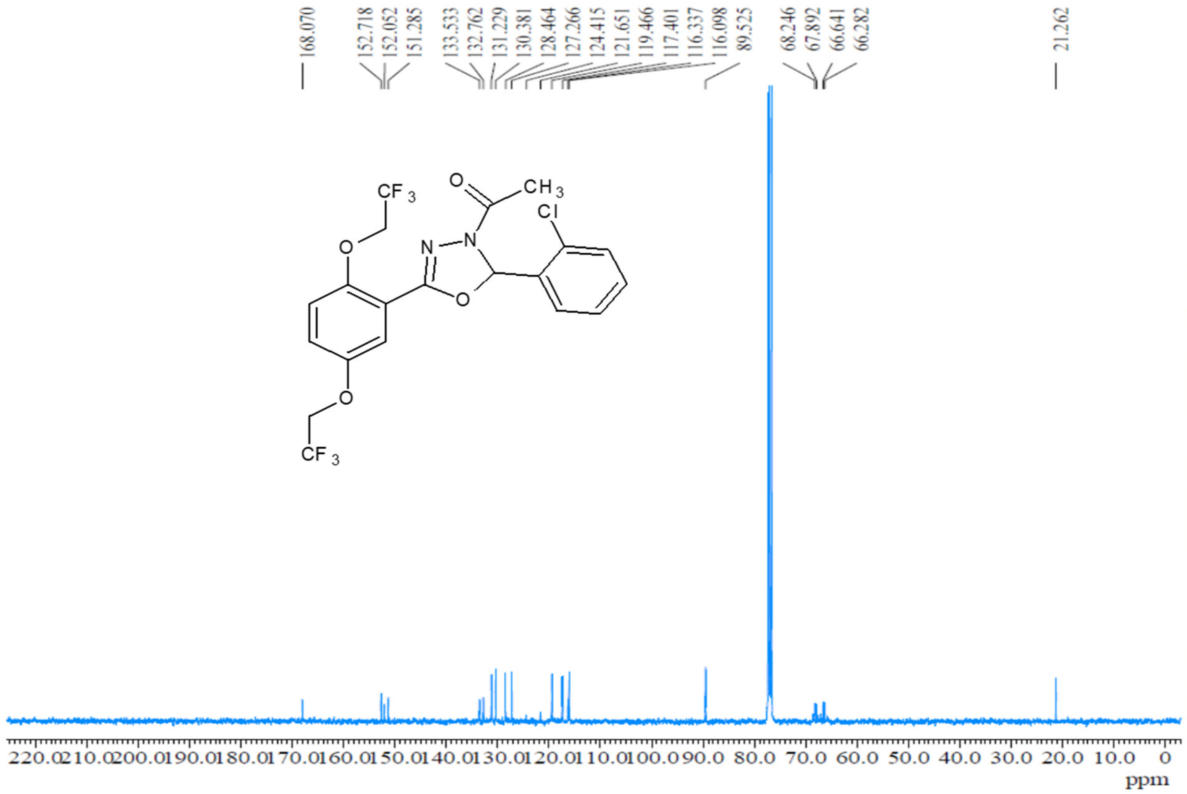


S.23: $^{13}$C NMR spectrum of 1-{5-[2,5-bis(2,2,2-trifluoroethoxy)phenyl]-2-(2-chloro phenyl)-1,3,4-oxadiazol-3(2*H*)-yl}ethanone(5f)

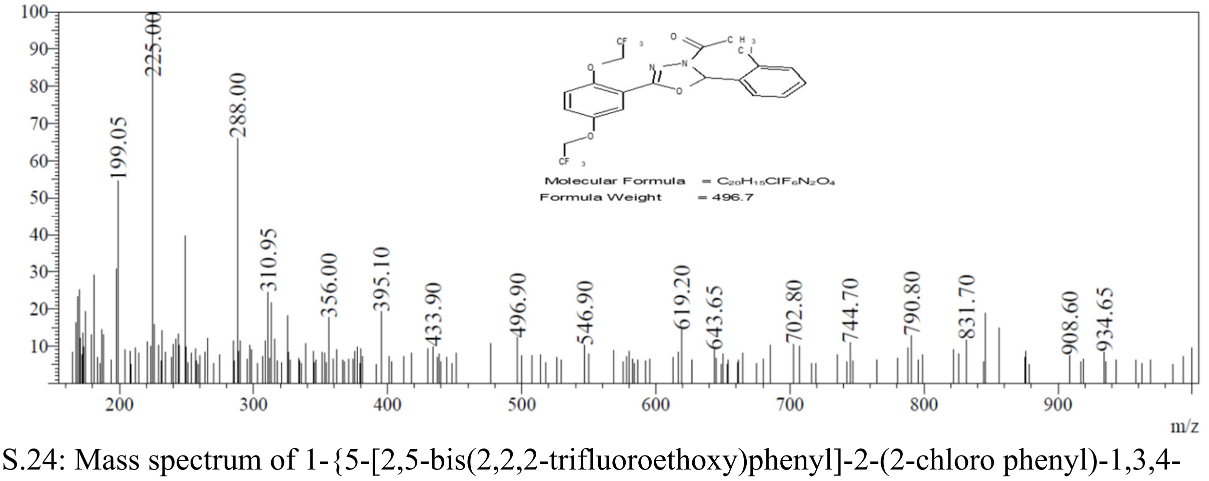


S.24: Mass spectrum of 1-{5-[2,5-bis(2,2,2-trifluoroethoxy)phenyl]-2-(2-chloro phenyl)-1,3,4-oxadiazol-3(2*H*)-yl}ethanone(5f)

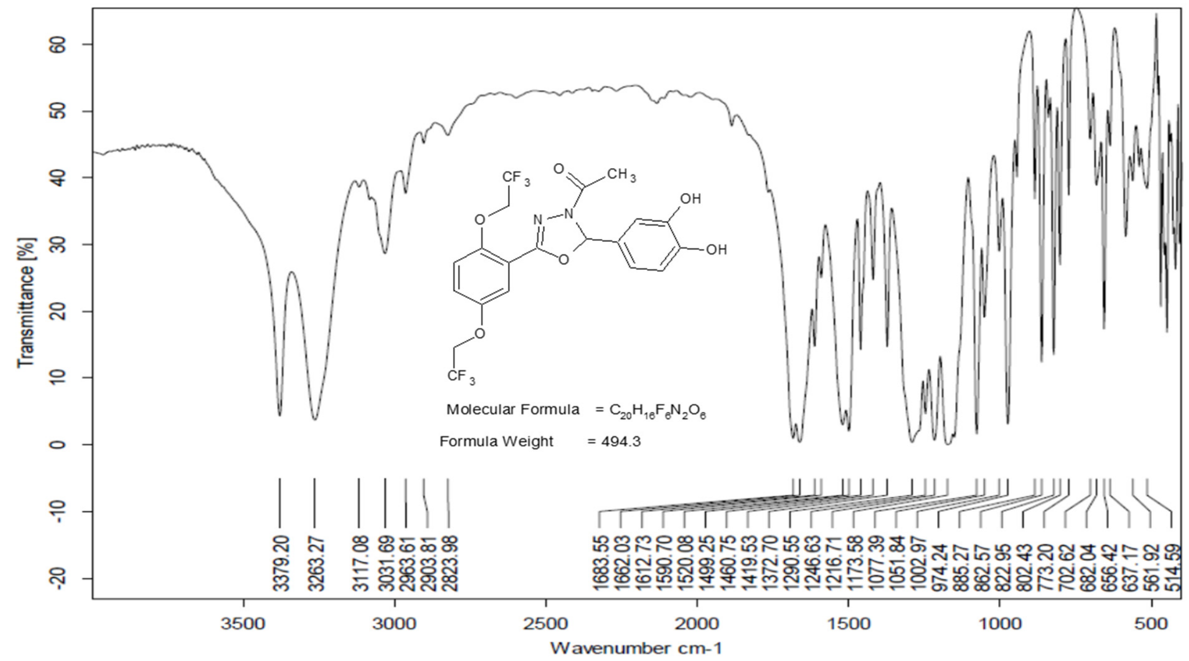


S.25: IR spectrum of 1-{5-[2,5-bis(2,2,2-trifluoroethoxy)phenyl]-2-(3,4-dihydroxy phenyl)-1,3,4-oxadiazol-3(2*H*)-yl}ethanone(5g)

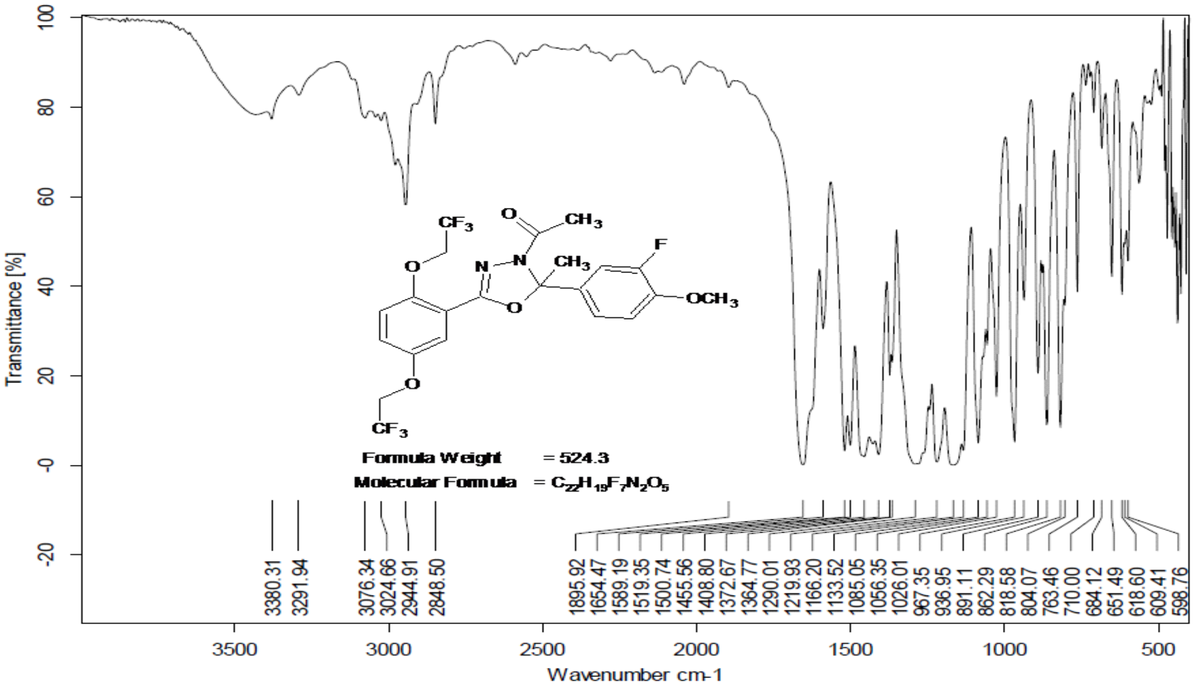


S.26: IR spectrum of 1-{5-[2,5-bis(2,2,2-trifluoroethoxy)phenyl]-2-(3-flouro-4-methoxy phenyl)-1,3,4-oxadiazol-3(2 *methyl*)-yl}ethanone(5i)

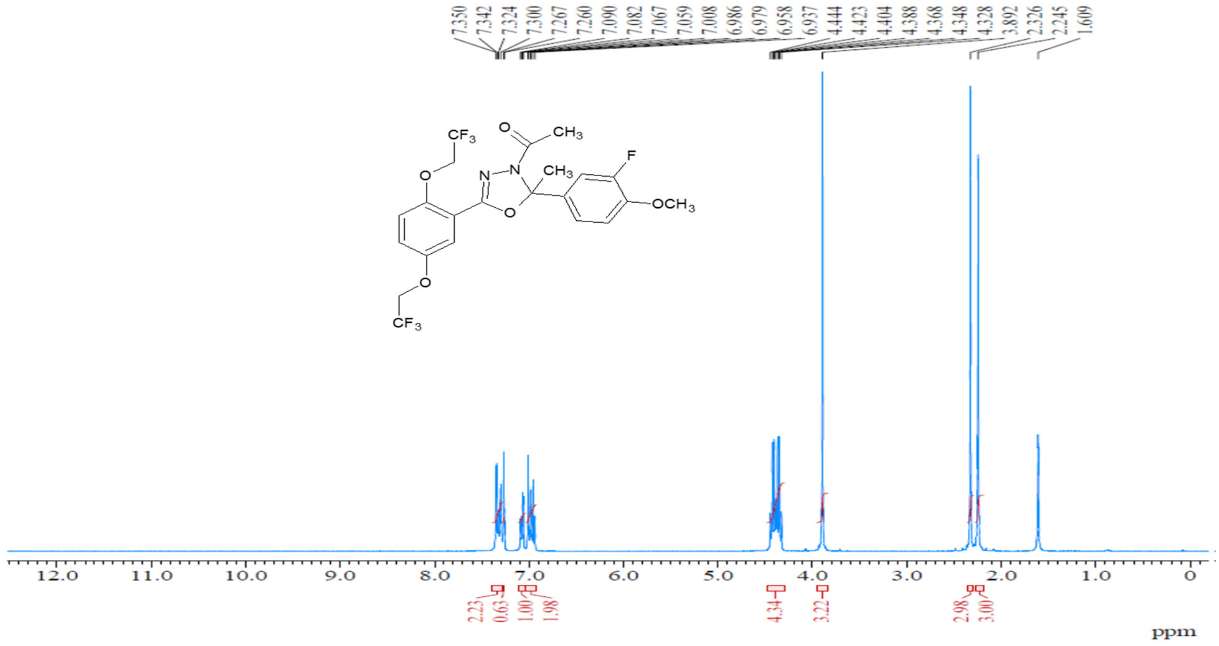

S.27: $^{1}$H NMR spectrum of 1-{5-[2,5-bis(2,2,2-trifluoroethoxy)phenyl]-2-(3-flouro-4-methoxy phenyl)-1,3,4-oxadiazol-3(2-methyl)-yl}ethanone(5i)

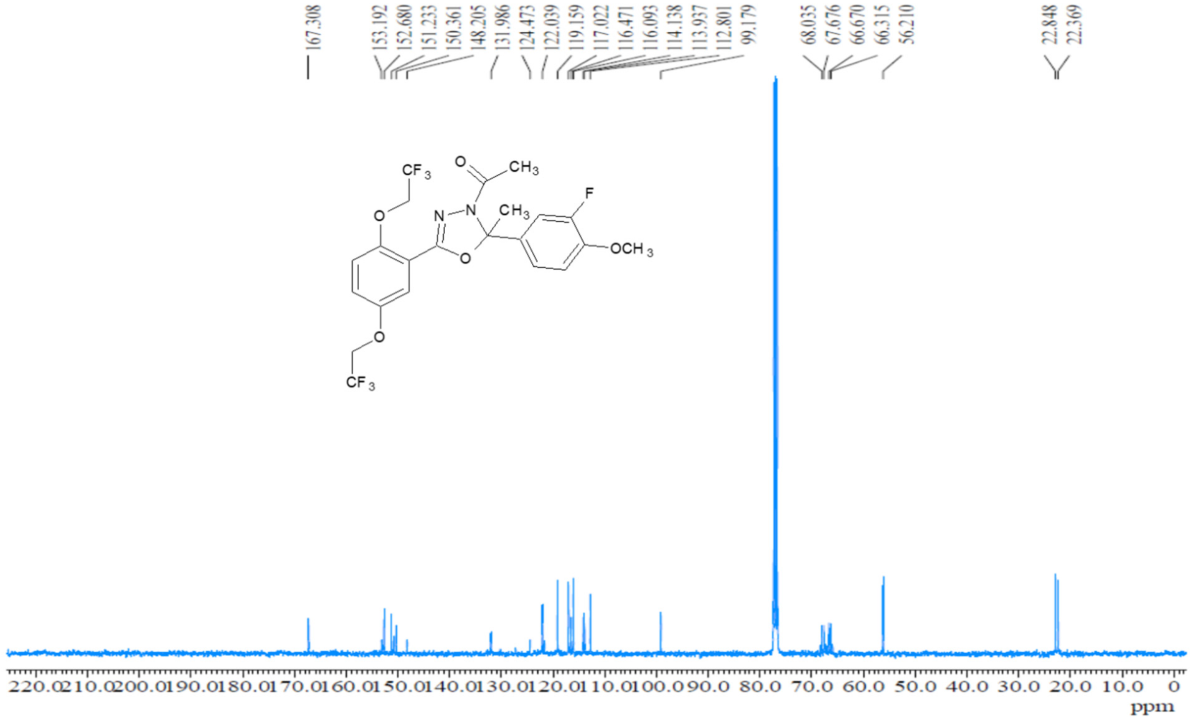

S.28: $^{13}$C NMR spectrum of 1-{5-[2,5-bis(2,2,2-trifluoroethoxy)phenyl]-2-(3-flouro-4-methoxy phenyl)-1,3,4-oxadiazol-3(2-methyl)-yl}ethanone(5i)

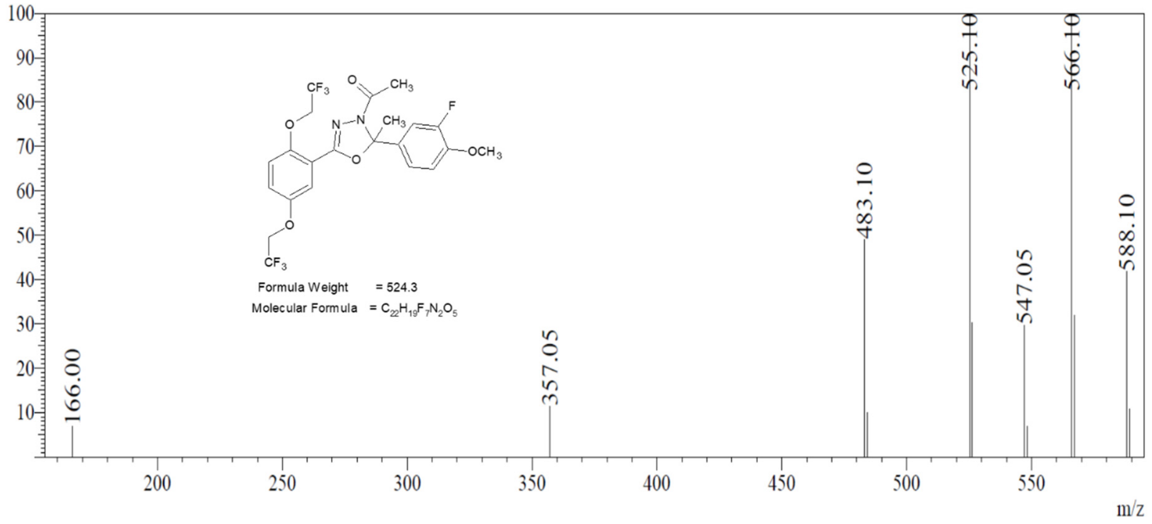


S.29: Mass spectrum of 1-{5-[2,5-bis(2,2,2-trifluoroethoxy)phenyl]-2-(3-flouro-4-methoxy phenyl)-1,3,4-oxadiazol-3(2-methyl)-yl}ethanone(5i)

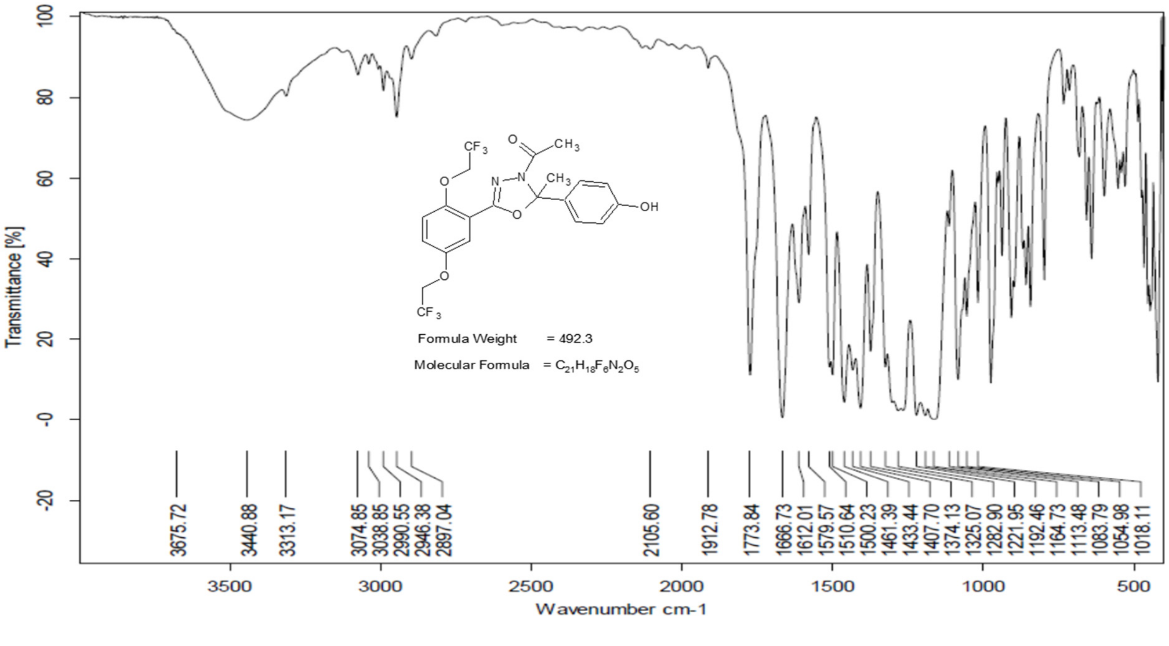


S.30: IR spectrum of 1-{5-[2,5-bis(2,2,2-trifluoroethoxy)phenyl]-2-(4-hydroxy phenyl)-1,3,4-oxadiazol-3(2 *methyl*)-yl}ethanone(5j)

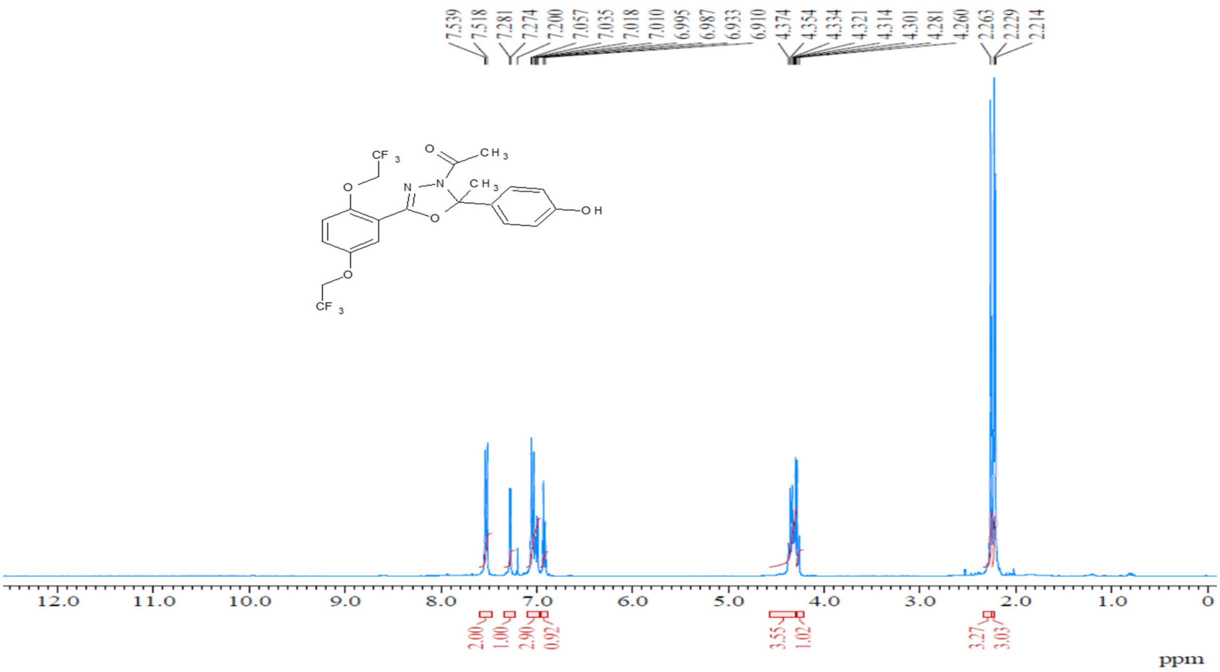


S.31: $^{1}$H NMR spectrum of 1-{5-[2,5-bis(2,2,2-trifluoroethoxy)phenyl]-2-(4-hydroxy phenyl)-1,3,4-oxadiazol-3(2-methyl)-yl}ethanone(5j)

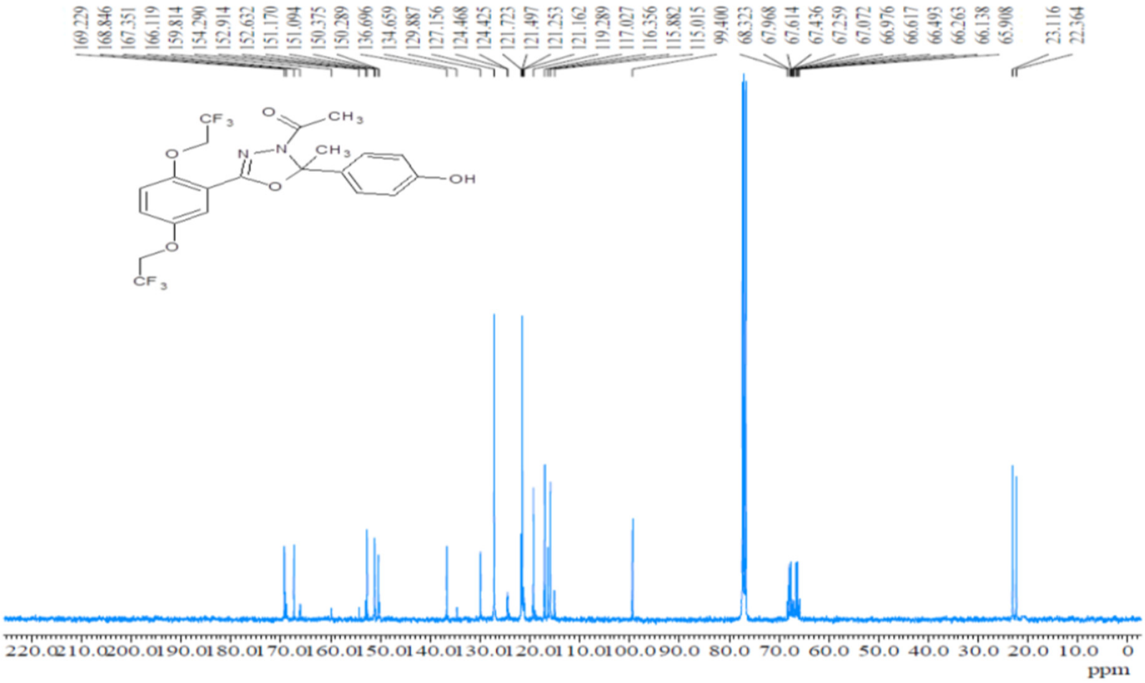


S.32: $^{13}$C NMR spectrum of 1-{5-[2,5-bis(2,2,2-trifluoroethoxy)phenyl]-2-(4-hydroxy phenyl)-1,3,4-oxadiazol-3(2-methyl)-yl}ethanone(5j)

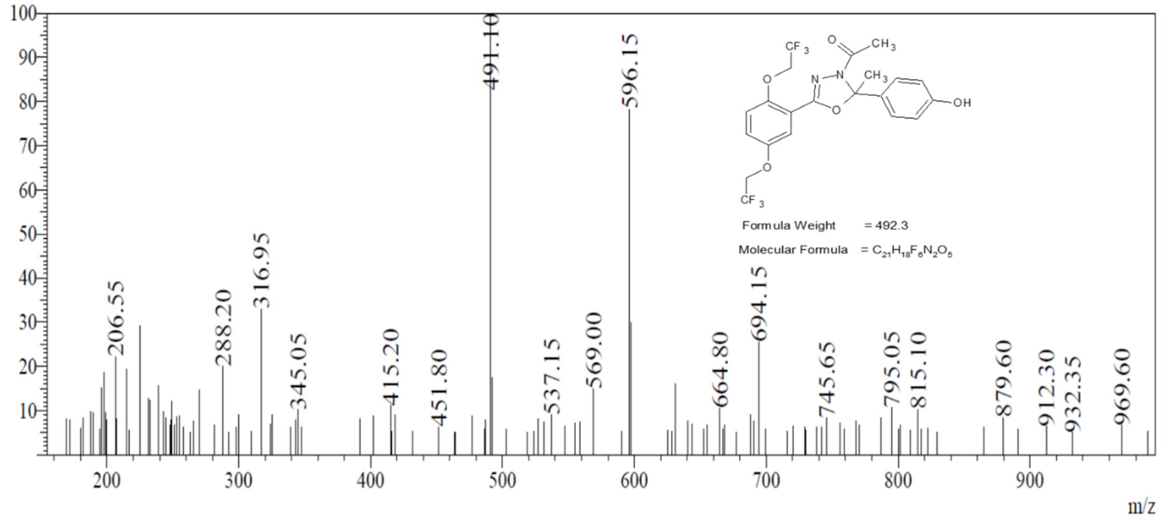


S.33: Mass spectrum of 1-{5-[2,5-bis(2,2,2-trifluoroethoxy)phenyl]-2-(4-hydroxy phenyl)-1,3,4-oxadiazol-3(2-methyl)-yl}ethanone(5j)

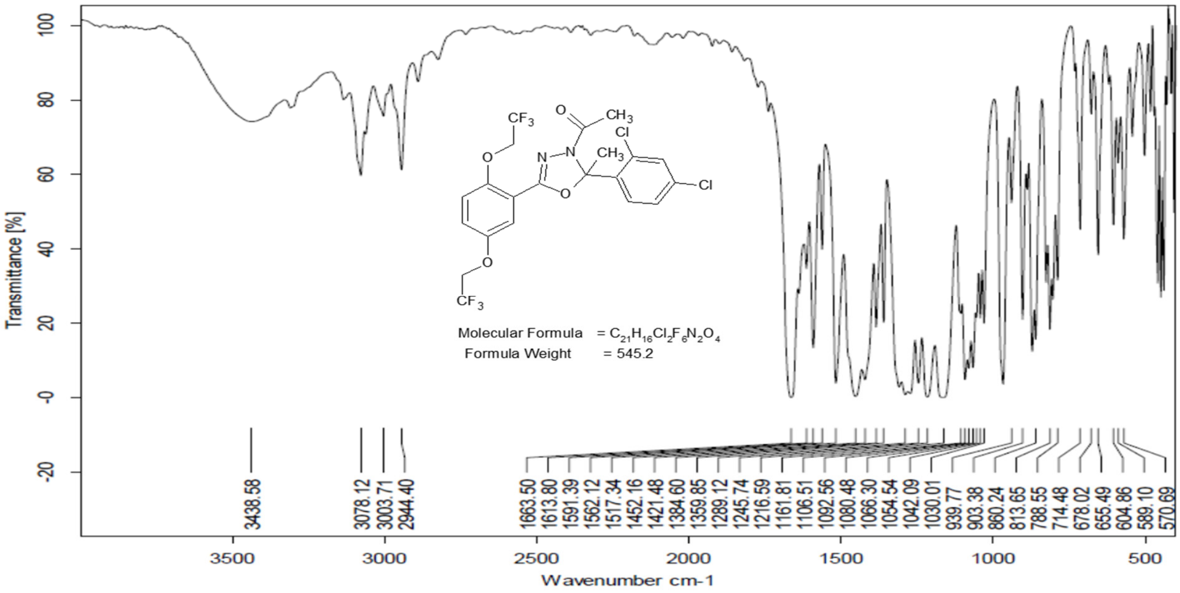


S.34: IR spectrum of 1-{5-[2,5-bis(2,2,2-trifluoroethoxy)phenyl]-2-(2,4-dichloro phenyl)-1,3,4-oxadiazol-3(2 *methyl*)-yl}ethanone(5k)

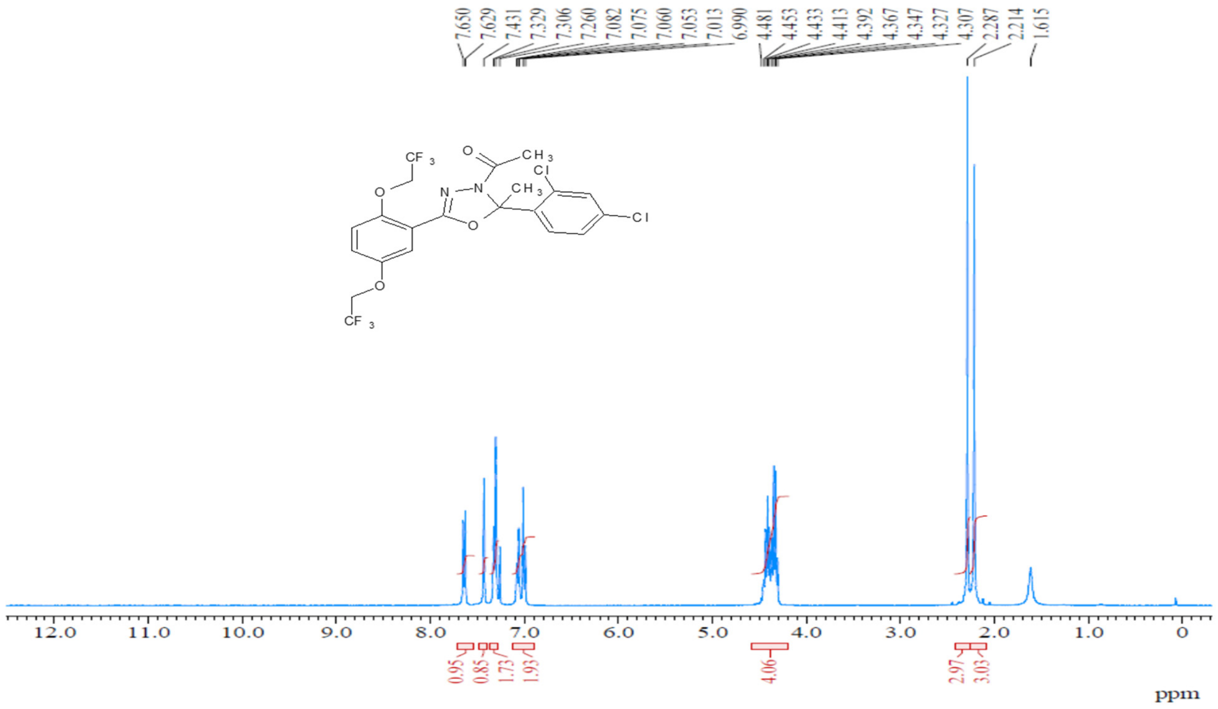

S.35: $^{1}$H NMR spectrum of 1-{5-[2,5-bis(2,2,2-trifluoroethoxy)phenyl]-2-(2,4-dichloro phenyl)-1,3,4-oxadiazol-3(2-methyl)-yl}ethanone(5k)

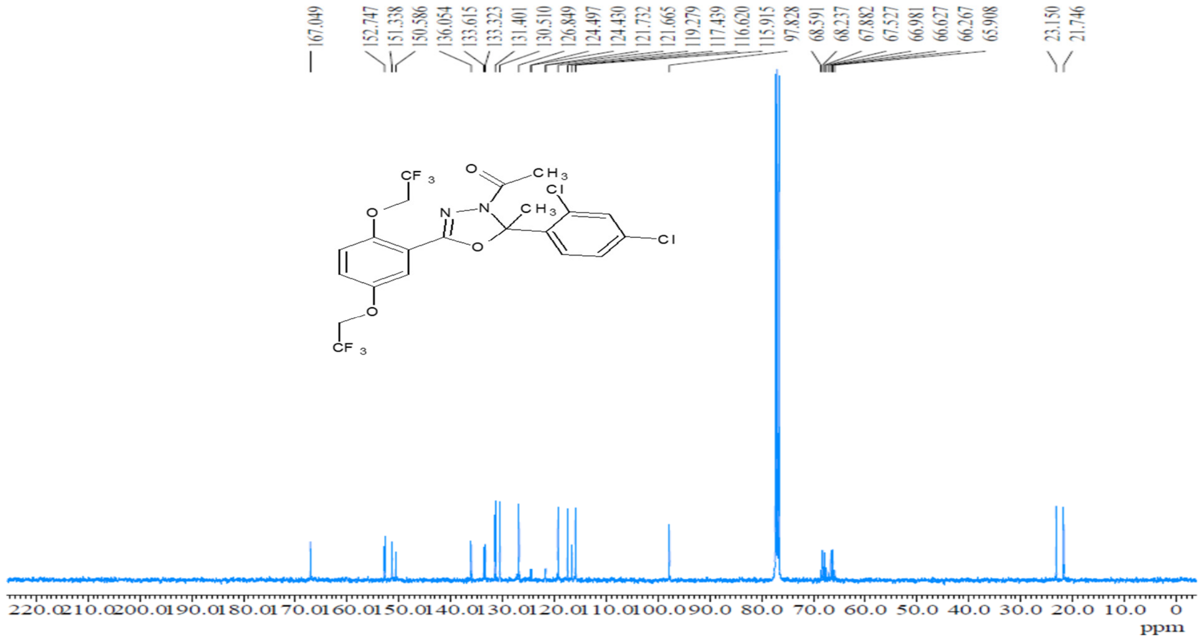

S.36: $^{13}$C NMR spectrum of 1-{5-[2,5-bis(2,2,2-trifluoroethoxy)phenyl]-2-(2,4-dichloro phenyl)-1,3,4-oxadiazol-3(2-methyl)-yl}ethanone(5k)

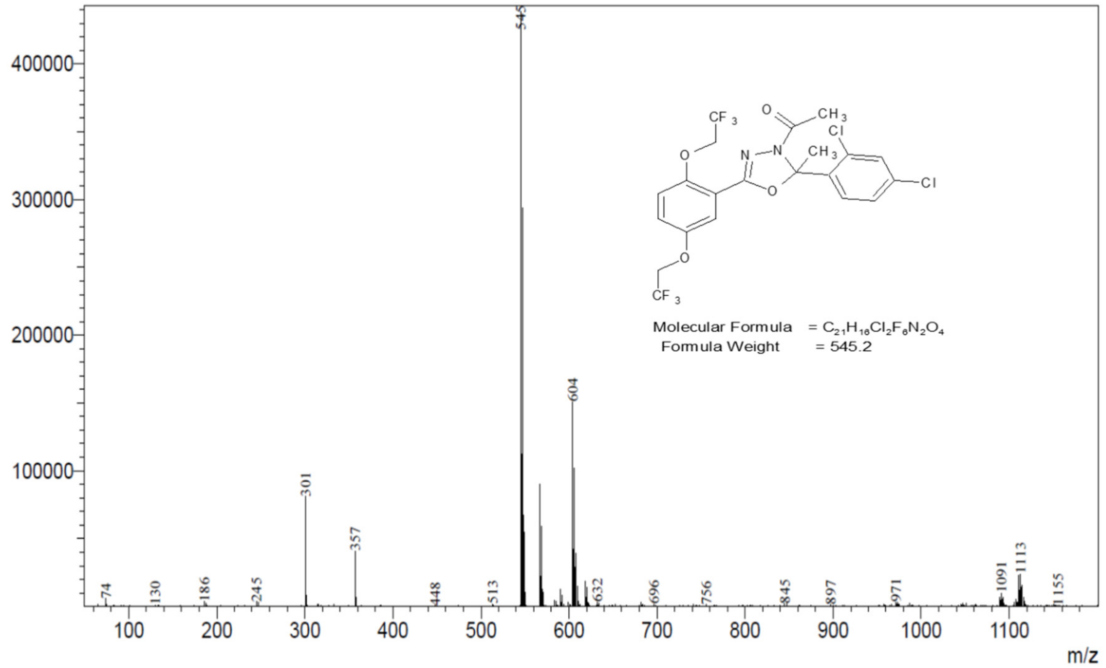


S.37: Mass spectrum of 1-{5-[2,5-bis(2,2,2-trifluoroethoxy)phenyl]-2-(2,4-dichloro phenyl)-1,3,4-oxadiazol-3(2-methyl)-yl}ethanone(5k)

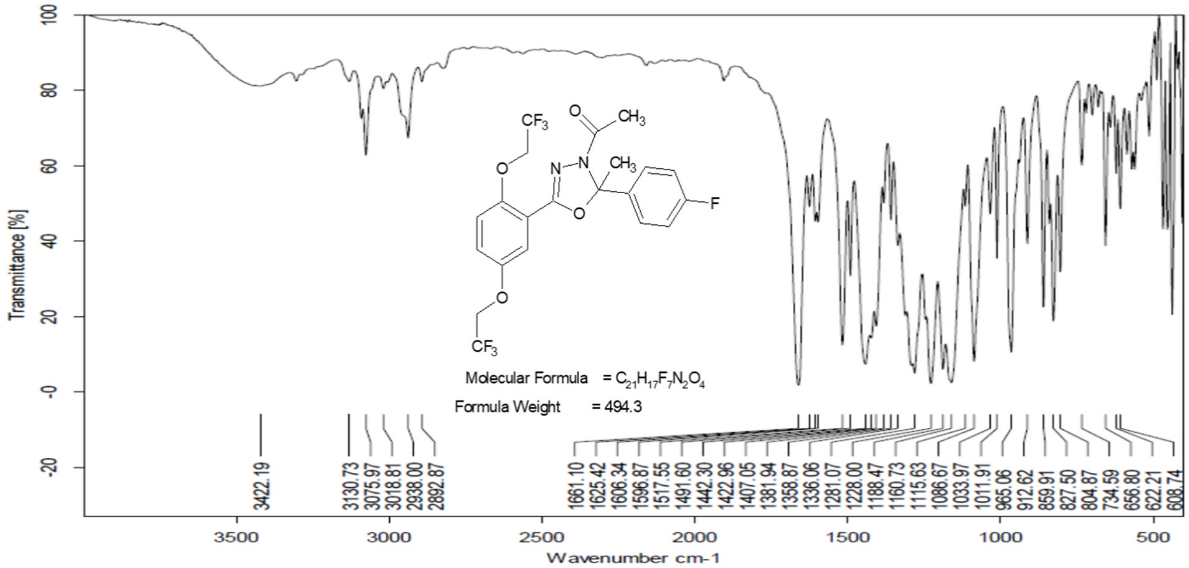


S.38: IR spectrum of 1-{5-[2,5-bis(2,2,2-trifluoroethoxy)phenyl]-2-(4-fluoro phenyl)-1,3,4-oxadiazol-3(2 *methyl*)-yl}ethanone(5l)

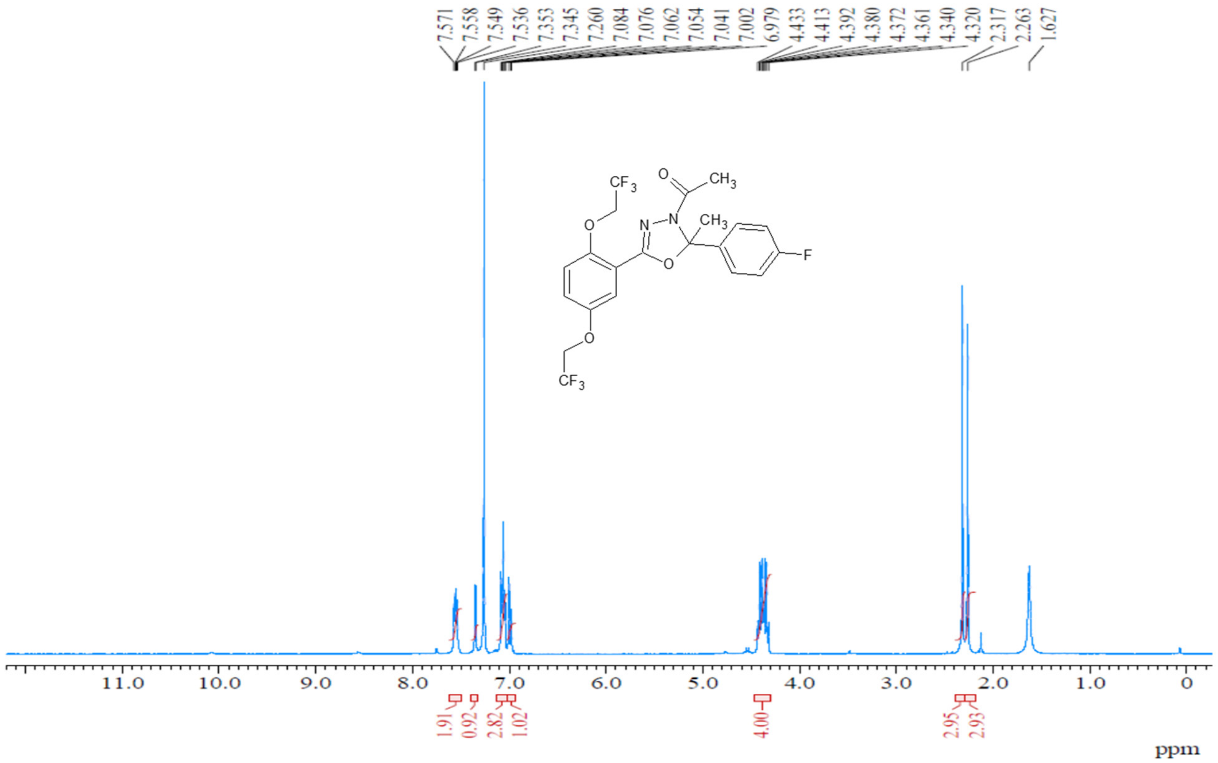

S.39: $^{1}$H NMR spectrum of 1-{5-[2,5-bis(2,2,2-trifluoroethoxy)phenyl]-2-(4-fluoro phenyl)-1,3,4-oxadiazol-3(2-methyl)-yl}ethanone(5l)

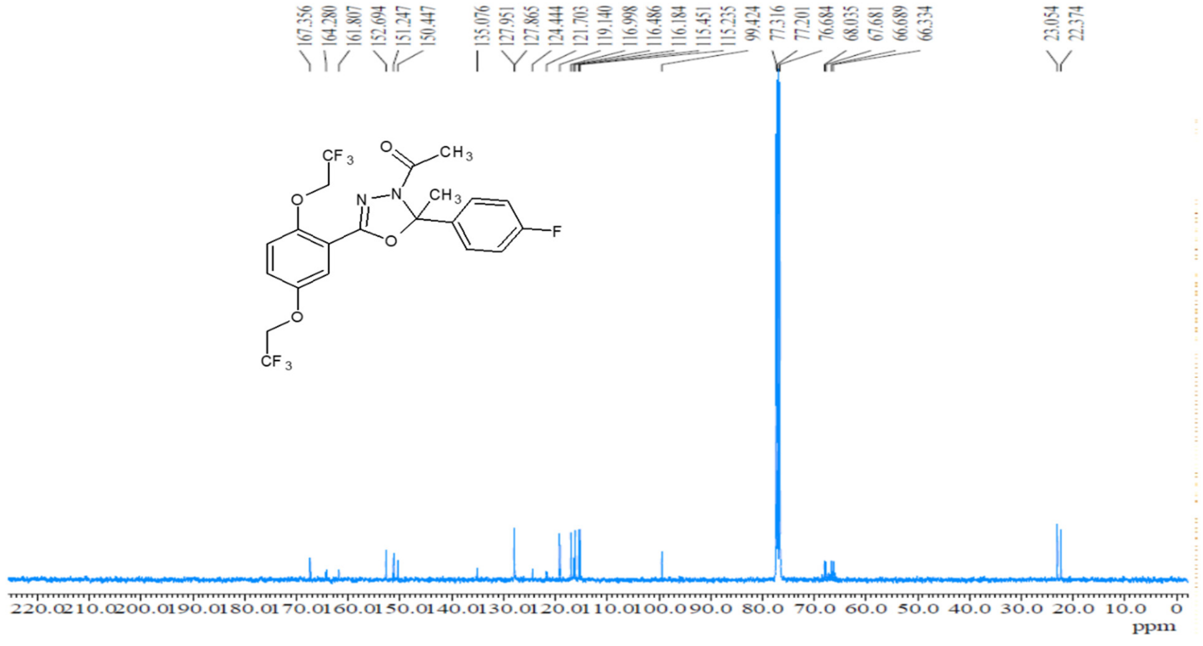

S.40: $^{13}$C NMR spectrum of 1-{5-[2,5-bis(2,2,2-trifluoroethoxy)phenyl]-2-(4-fluoro phenyl)-1,3,4-oxadiazol-3(2-methyl)-yl}ethanone(5l)

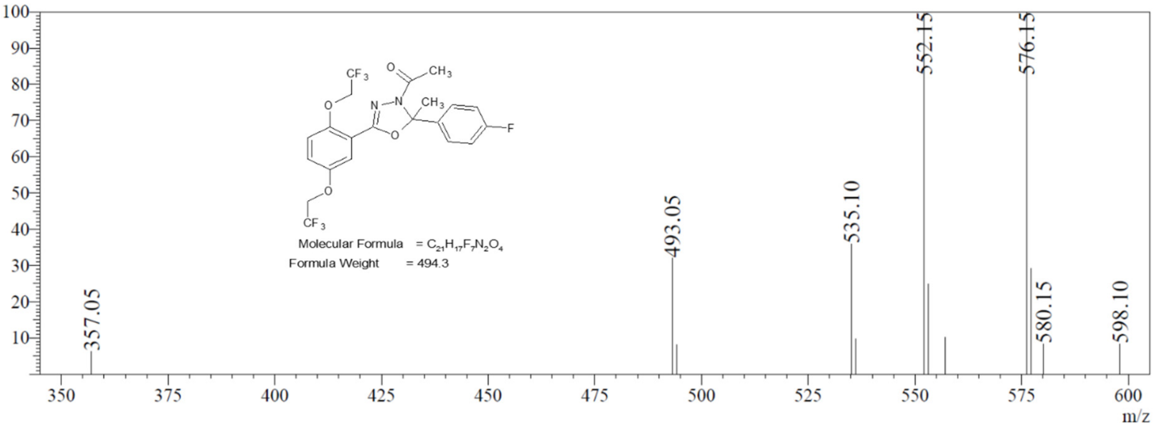


S.41: Mass spectrum of 1-{5-[2,5-bis(2,2,2-trifluoroethoxy)phenyl]-2-(4-fluoro phenyl)-1,3,4-oxadiazol-3(2-methyl)-yl}ethanone(5l)

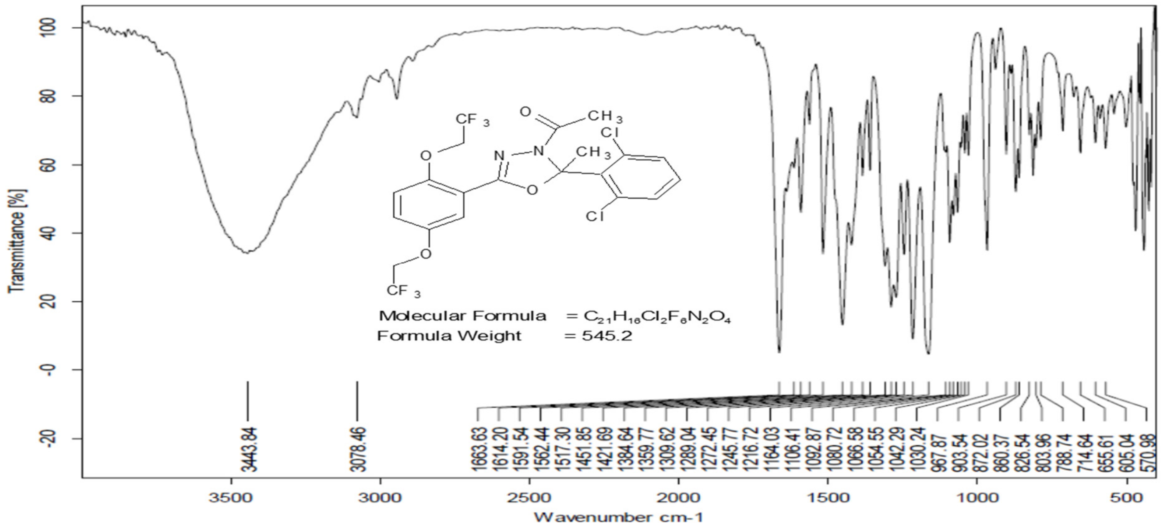


S.42: IR spectrum of 1-{5-[2,5-bis(2,2,2-trifluoroethoxy)phenyl]-2-(2,6-dichloro phenyl)-1,3,4-oxadiazol-3(2-methyl)-yl}ethanone(5m)

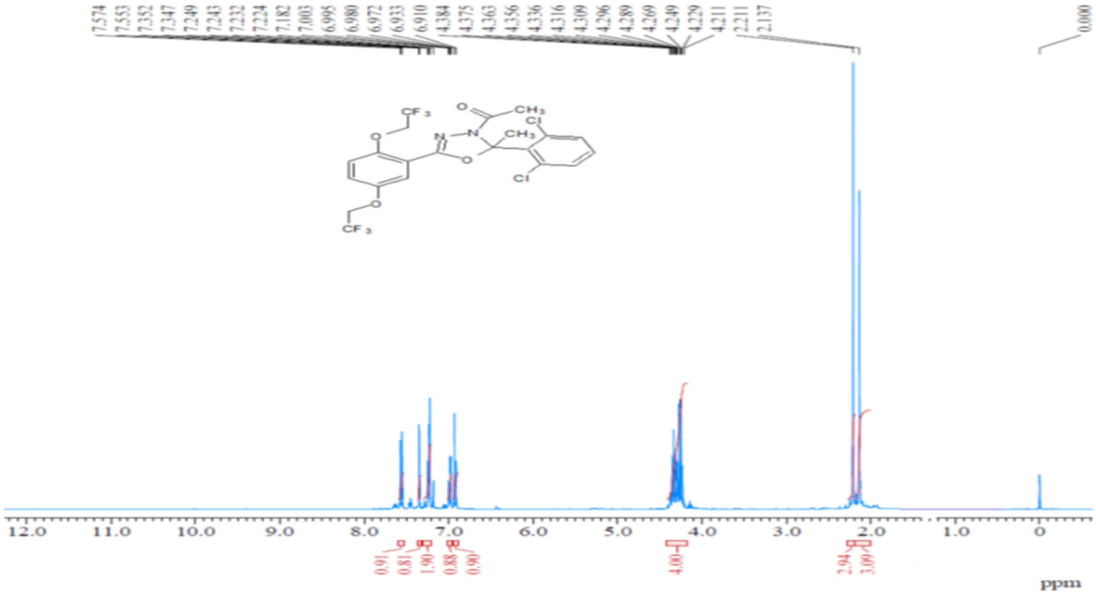


S.43: $^{1}$H NMR spectrum of 1-{5-[2,5-bis(2,2,2-trifluoroethoxy)phenyl]-2-(2,6-dichloro phenyl)-1,3,4-oxadiazol-3(2-methyl)-yl}ethanone(5m)

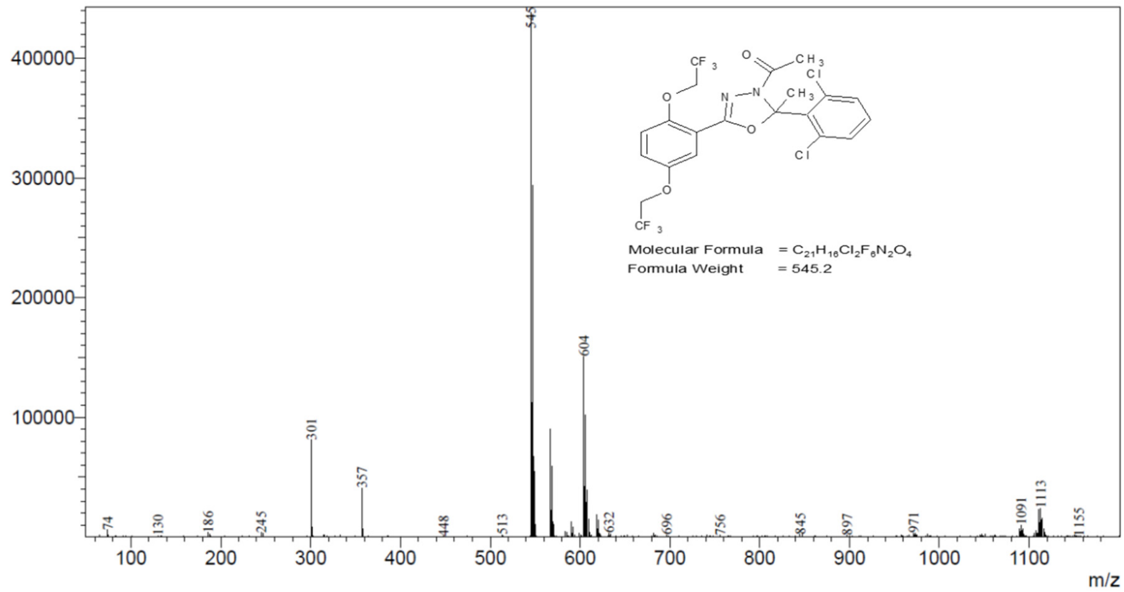


S.44: Mass spectrum of 1-{5-[2,5-bis(2,2,2-trifluoroethoxy)phenyl]-2-(2,6-dichloro phenyl)-1,3,4-oxadiazol-3(2-methyl)-yl}ethanone(5m)

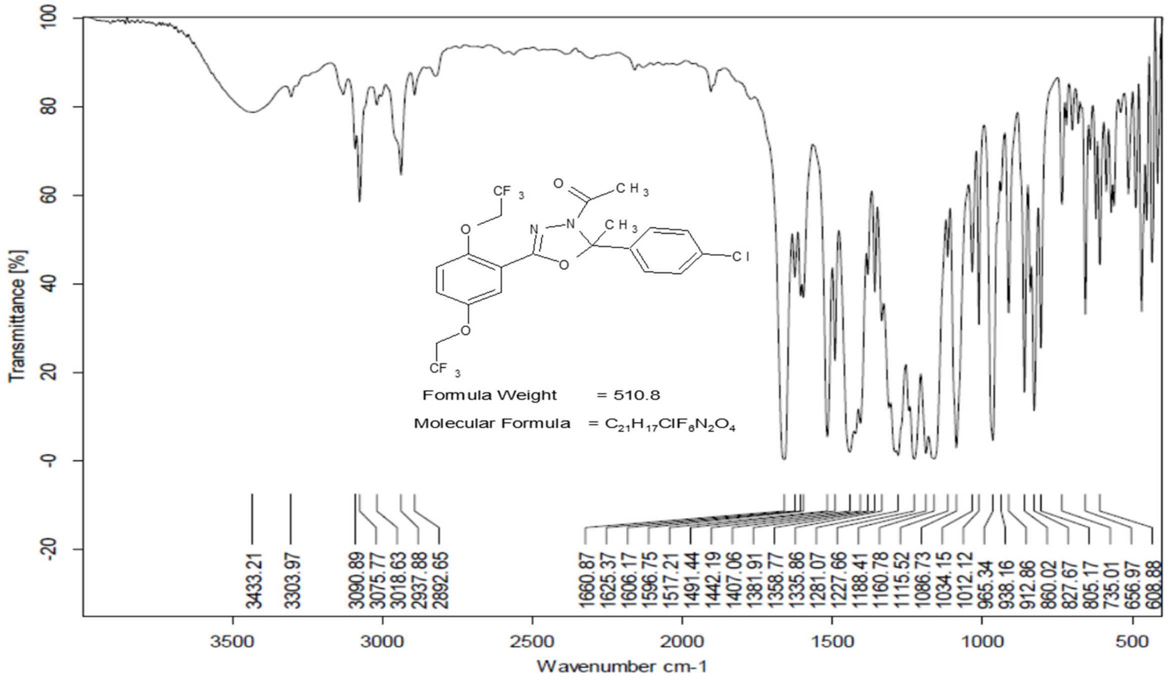


S.45: IR spectrum of 1-{5-[2,5-bis(2,2,2-trifluoroethoxy)phenyl]-2-(4-chloro phenyl)-1,3,4-oxadiazol-3(2-methyl)-yl}ethanone(5n)

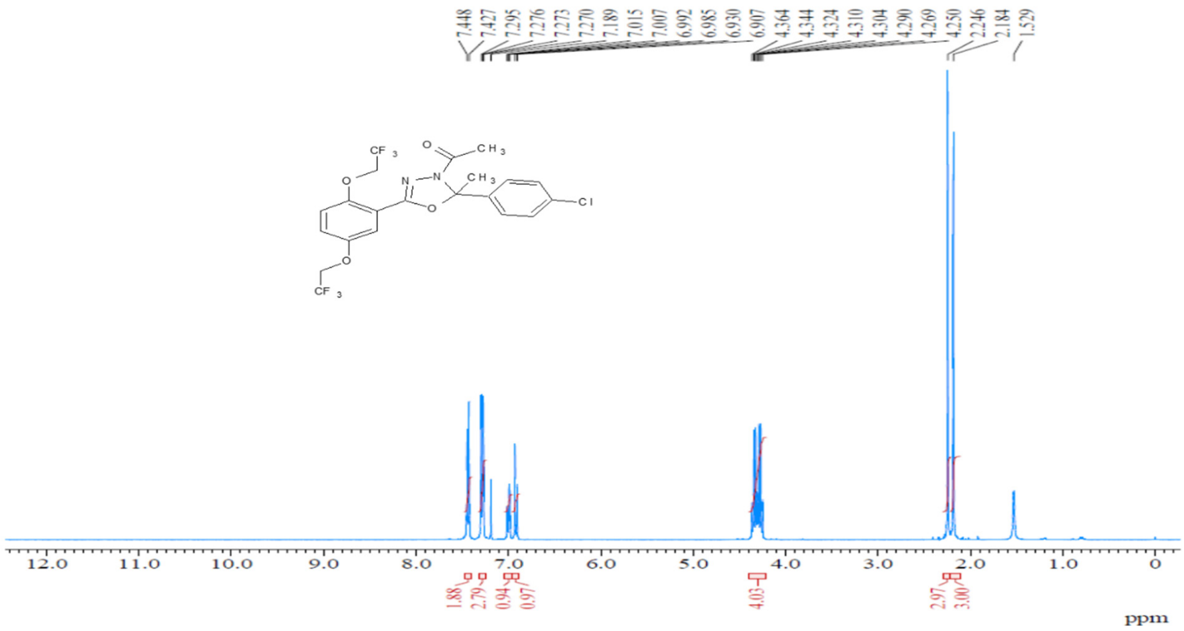


S.46: $^{1}$H NMR spectrum of 1-{5-[2,5-bis(2,2,2-trifluoroethoxy)phenyl]-2-(4-chloro phenyl)-1,3,4-oxadiazol-3(2-methyl)-yl}ethanone(5n)

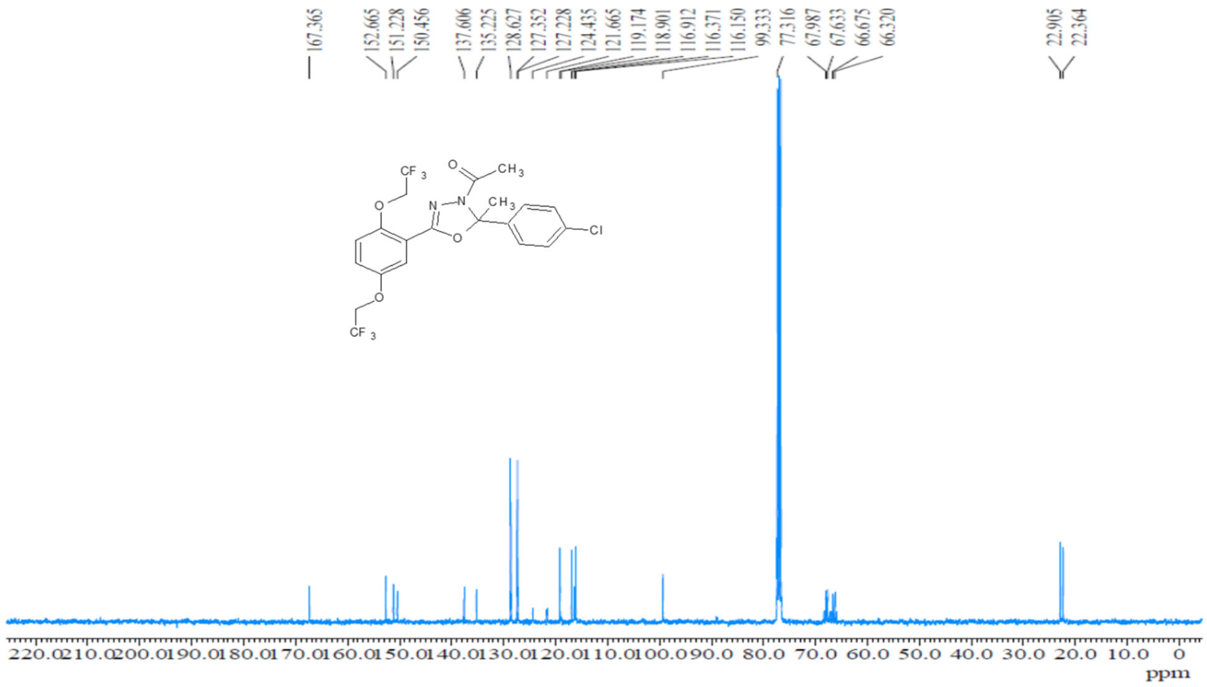


S.47: $^{13}$C NMR spectrum of 1-{5-[2,5-bis(2,2,2-trifluoroethoxy)phenyl]-2-(4-chloro phenyl)-1,3,4-oxadiazol-3(2-methyl)-yl}ethanone(5n)

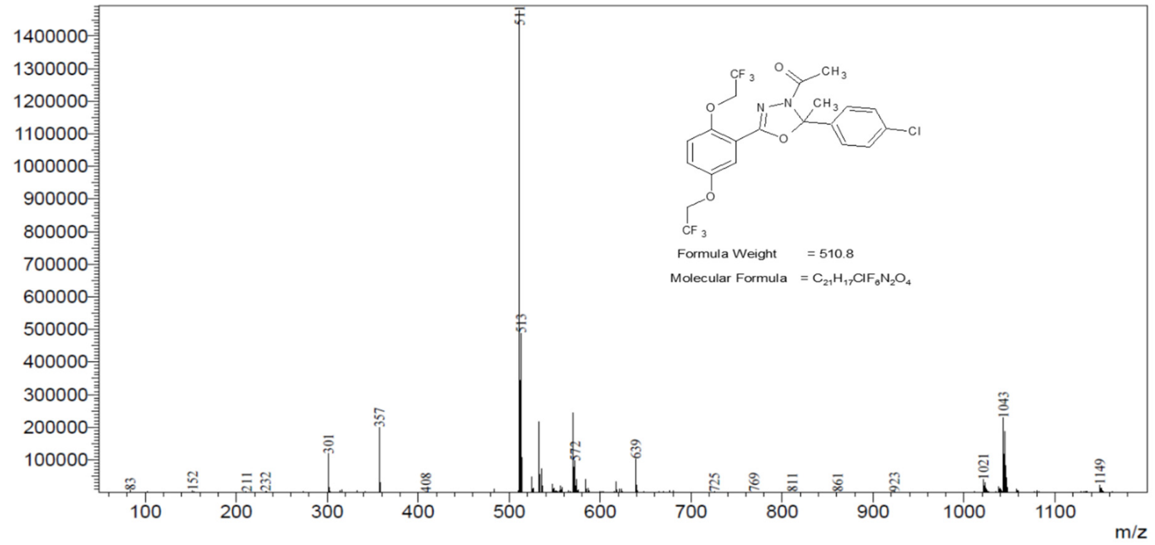


S.48: Mass spectrum of 1-{5-[2,5-bis(2,2,2-trifluoroethoxy)phenyl]-2-(4-chloro phenyl)-1,3,4-oxadiazol-3(2-methyl)-yl}ethanone(5n)
